# Grading Canine Benign Prostatic Hyperplasia: A Survey of Veterinary Practice and the Development of a Web-Based Symptom Index

**DOI:** 10.3390/ani16182833

**Published:** 2026-09-09

**Authors:** Daniele Zambelli, Giulia Ballotta, Diego Bucci, Lucia Pagliuca, Marco Cunto

**Affiliations:** 1 Department of Veterinary Medical Sciences, University of Bologna, Ozzano dell’Emilia, 40064 Bologna, Italy; daniele.zambelli@unibo.it (D.Z.); diego.bucci3@unibo.it (D.B.); marco.cunto@unibo.it (M.C.); 2 Independent Researcher, 40100 Bologna, Italy; luciapagliuca.vet@gmail.com

**Keywords:** BPH grading, prostatic disorder, BPH symptoms, dog, symptom-based assessment

## Abstract

Benign prostatic hyperplasia (BPH) affects more than 95% of intact male dogs, yet most remain asymptomatic until prostatic enlargement causes clinical signs. Standardised assessment of BPH severity is therefore needed to improve clinical decision-making and enable comparable research outcomes. This study aimed to investigate current diagnostic and therapeutic approaches to canine BPH and to develop and assess a web-based application implementing the Zambelli Symptom Index (ZSI), an established symptom-based scoring system for BPH severity. A voluntary questionnaire was completed by veterinary practitioners to assess current clinical practice, and agreement between the web-based and reference paper-based ZSI classifications was evaluated. The survey revealed considerable variability in the recognition of clinical signs, assessment of BPH severity, and selection of treatment strategies, despite broad agreement on the diagnostic value of ultrasonography and the need for further investigation of prostatic enlargement. The web-based ZSI showed very high agreement with the reference classification. These findings demonstrate that the ZSI can be implemented digitally without materially affecting severity classification. The web-based tool may improve the standardisation and reproducibility of BPH assessment in clinical practice and research. Further studies are warranted to evaluate interobserver variability, clinical validity, and responsiveness to treatment.

## Simple Summary

Benign prostatic hyperplasia (BPH) is common in intact male dogs, but many affected dogs show no clinical signs until the prostate enlarges. Assessing BPH severity can be challenging, as veterinarians may differ in recognising clinical signs and making treatment decisions. This study investigated current approaches to diagnosing and managing canine BPH and evaluated a web-based tool to standardise disease severity assessment. The survey revealed considerable variability among veterinarians in recognising clinical signs, assessing BPH severity, and selecting treatment strategies, despite broad agreement on the importance of ultrasonography and further investigation of prostatic enlargement. The web-based tool, based on the Zambelli Symptom Index, showed very high agreement with the original paper-based classification. These results indicate that the Zambelli Symptom Index can be reliably implemented in a web-based format. The tool could help veterinarians assess BPH severity more consistently and support standardised clinical decision-making.

## 1. Introduction

Benign prostatic hyperplasia (BPH) is the most common prostatic disorder in sexually intact male dogs and is an age-related, androgen-dependent condition in which alterations in the androgen-to-estrogen balance may contribute to prostatic growth [1]. It develops spontaneously in men, several non-human primates (including chimpanzees and macaques), and dogs, following an age-related, androgen-dependent pattern [2]. With nearly all individuals developing the disorder over time, studies indicate that more than 95% of intact male dogs are affected by 9 years of age [3]. However, this high prevalence should not be interpreted as meaning that 95% of older intact dogs are clinically ill and therefore require treatment.

Although prostatic enlargement is a characteristic finding of BPH, prostate size does not necessarily reflect clinical severity, and prostatomegaly alone should not be used to identify dogs with clinically relevant disease [1]. In a substantial proportion of ageing intact dogs, prostatic hyperplasia remains asymptomatic and may reasonably be regarded as an androgen-dependent manifestation of ageing. BPH-related clinical signs are relatively common, particularly in dogs older than 6 years, but typically develop only when hyperplasia progresses sufficiently to cause clinically relevant changes [3,4]. Therefore, histological or ultrasonographic evidence of BPH should be distinguished from clinically significant disease. The presence of prostatic enlargement alone should not constitute an indication for treatment; rather, therapeutic decisions should be guided by the presence and severity of clinical signs, associated complications, impairment of reproductive function, and the individual dog’s welfare [1,5]. Accordingly, treatment should be considered primarily when BPH is associated with clinically relevant clinical signs or complications [6].

Among symptomatic dogs, the most commonly reported signs include sanguineous preputial or urethral discharge, haematuria, and haemospermia. When marked prostatic enlargement or large prostatic cysts are present, secondary gastrointestinal or perineal complications, such as constipation, rectal tenesmus with ribbon-shaped faeces, dyschezia, or perineal hernia, may occur. Dysuria or urinary incontinence is less common and typically results from urethral compression. In breeding males, BPH may contribute to subfertility, often associated with substantial erythrocytosis in the prostatic fluid [5].

A standardised classification of BPH severity is essential to minimise the subjectivity inherent in clinical evaluation and to support appropriate therapeutic decision-making. Symptom assessment is therefore central to BPH management and should be based on objective scoring tools that provide a reproducible measure of clinical severity and enable consistent monitoring of treatment response over time.

In human medicine, the International Prostate Symptom Score (IPSS) is recognised as the gold standard for quantifying symptoms and is widely used in BPH research [7]. Roehrborn et al. [8] reviewed international guidelines for BPH, noting that most recommend structured symptom assessment. The IPSS, derived from the American Urological Association Symptom Index (AUA-SI), is the most commonly used instrument [9]. Across these guidelines, therapeutic recommendations are primarily based on symptom presence and severity, as well as patient-reported discomfort. Watchful waiting suits mild symptoms or minimal impact on quality of life. Medical therapy is indicated for moderate symptoms or interference with daily life. Surgery is reserved for severe cases, complications, or treatment failure [7,8].

In veterinary medicine, however, assessment of symptomatic BPH has traditionally relied largely on subjective clinical evaluation, limiting consistency in the classification and comparison of affected dogs. To address this limitation, Zambelli et al. introduced the first canine-specific BPH Symptom Index in 2012, inspired by the IPSS [10]. The Zambelli Symptom Index (ZSI) provides a standardised assessment of clinical severity based on BPH-related symptoms rather than prostate size. The index considers the presence and severity of BPH-related clinical signs, including urinary and gastrointestinal disturbances and systemic manifestations, which are combined to assign dogs to three levels of clinical severity: low (grade 1), moderate (grade 2), and high (grade 3). In the original development study, the model correctly predicted the assigned severity category for 97.3% of the 373 included dogs. The ZSI was developed to quantify the severity of clinical signs associated with canine BPH. The ZSI has subsequently been used in clinical trials to standardise case selection, improve comparability between treatment groups, and detect differences in treatment outcomes [1,11,12].

The present study had two primary objectives: (1) to investigate current approaches used by veterinary practitioners for the clinical evaluation of canine benign prostatic hyperplasia (BPH), through a questionnaire, and (2) to develop and evaluate a web-based application for the standardised grading of canine BPH severity. The questionnaire investigated the clinical history, clinical signs, diagnostic findings, and reproductive parameters routinely considered when assessing BPH severity and determining the need for therapeutic intervention. This survey was intended to identify variability among clinicians and areas where greater standardisation may be warranted. The web-based application was designed to facilitate data sharing among clinicians managing canine BPH by providing a simple, consistent, and objective scoring framework, as previously recommended by Zambelli et al. [10].

## 2. Materials and Methods

### 2.1. Study 1: Webinar-Based Questionnaire for Veterinary Practitioners’ Evaluation of the BPH Approach

#### 2.1.1. Study Design and Questionnaire Development

An anonymous questionnaire was administered via the Slido audience-interaction platform during the “Reproduction” webinar, an educational event focused on male canine reproductive medicine, held in May 2026. The webinar was conducted entirely online in Italian and was part of a non-academic educational series open to all practising veterinarians, regardless of professional interests and backgrounds. The questionnaire was developed by Professor Daniele Zambelli, who also conducted the webinary, to assess participants’ clinical approaches to canine prostatic disease, with particular emphasis on benign prostatic hyperplasia (BPH). It comprised single-answer multiple-choice questions only and was organised into four main domains: (1) screening practices, (2) diagnostic work-up, (3) assessment of BPH severity, and (4) management of BPH. Before administration, the questionnaire was reviewed by the authors, who have expertise in canine reproduction and prostatic disease, including the expert who conducted the webinar, to assess the relevance, clarity, and consistency of the questions.

To minimise response bias, all questions, including those based on clinical case scenarios, were presented before any explanation or discussion of the corresponding topics during the webinar. The sequence of questions was predetermined to prevent information provided during the webinar from influencing subsequent responses. Each question was presented only once during the live webinar and could be answered only during its presentation; participants could not exit and re-enter the questionnaire to submit a second response. Participants could select only one response per question. Participation was voluntary and anonymous, and respondents were not required to answer every question.

#### 2.1.2. First Section—Screening Practices

The first group of questions investigated veterinarians’ routine screening practices for canine prostatic disease. Participants were asked whether they routinely screened only intact dogs or both intact and neutered dogs. Additional questions addressed the age at which screening should begin and the recommended screening interval, with response options ranging from annual screening starting at puberty to screening limited to senior dogs.

Participants were also asked to indicate the screening method routinely used in clinical practice. Options included no routine screening, digital rectal examination (DRE), serum canine prostate-specific esterase (CPSE) measurement, ultrasonography, or predefined combinations of these methods.

#### 2.1.3. Second Section—Diagnostic Work-Up

The second group of questions evaluated the diagnostic approach for dogs with suspected prostatic disease. Participants were asked to identify the most commonly observed clinical sign in affected dogs from among constipation or tenesmus, including ribbon-like stools; serosanguineous, haemorrhagic, or purulent urethral discharge; haematuria; and dysuria, urinary retention, or urinary obstruction. A response option listing all the clinical signs was also provided. Respondents were then asked to select the preferred initial diagnostic work-up for an intact dog with prostatic enlargement. The proposed investigations included ultrasonography, cytological examination and bacteriological culture, biochemical analysis of cyst fluid when a prostatic cyst was present, or a combination of all these investigations.

A further question examined the techniques used to obtain biological samples when prostatic disease was suspected. The response options included prostatic massage, fine-needle aspiration (FNA) of the prostatic parenchyma and any cystic lesions, seminal fluid collection, urine collection by cystocentesis, voided urine collection, and predefined combinations of these sampling techniques. Some combined options were formulated according to the suspected diagnosis, the invasiveness of the procedure, and the perceived risk associated with sampling in cases of suspected infection or neoplasia.

#### 2.1.4. Third Section—Assessment of BPH Severity

The third group evaluated respondents’ ability to assess BPH severity and the criteria used to assign a severity grade. This section comprised four clinical case scenarios (Figures S1–S4). For each case, participants were provided with the dog’s signalment (breed, age, and body weight), relevant clinical signs, serum CPSE concentration, ultrasonographic prostate dimensions, expected and measured prostatic volumes, and representative ultrasonographic images.

Based on the information provided, respondents were asked to classify each case into one of five categories: normal prostate, asymptomatic BPH, mild BPH, moderate BPH, or severe BPH. For each case, the ZSI classification was used as the reference severity grade.

After completing the four case assessments, participants were asked to identify the primary criterion used to grade BPH severity. The response options comprised clinical signs; ultrasonographic findings, including prostate size, parenchymal appearance, and the presence of cystic lesions; serum CPSE concentration; a combination of serum CPSE concentration and ultrasonographic findings; or a combination of Digital Rectal Examination (DRE) findings and serum CPSE concentration.

#### 2.1.5. Fourth Section—Management of BPH

The fourth group of questions examined decision-making regarding the management and treatment of canine BPH. Participants were first asked how they would manage an intact dog in which prostatic enlargement had been detected by DRE. The available responses included performing further diagnostic investigations, recommending orchiectomy, initiating medical treatment, or recommending no intervention if the dog was asymptomatic.

Participants were also asked which medications they currently used or had previously used to treat BPH. The response options included finasteride, osaterone acetate, deslorelin, cyproterone acetate or flutamide, and predefined combinations of these treatments.

Therapeutic decision-making was further assessed using four clinical case scenarios (Figures S5–S8). As in the severity-assessment section, each case included the dog’s signalment, clinical signs, serum CPSE concentration, ultrasonographic prostate dimensions, expected and measured prostatic volumes, and representative ultrasonographic images. Based on these data, participants were asked to select the therapeutic approach they considered most appropriate. The management strategy derived from the ZSI assessment served as the reference approach for each case.

#### 2.1.6. Data Analysis

Responses were summarised using descriptive statistics. As completion of individual questionnaire items was optional, the number of valid responses varied by item. For each questionnaire item, categorical variables are reported as absolute frequencies and percentages of respondents selecting each response option. Response distributions were analysed to characterise current veterinary practices for the diagnosis, grading, treatment, and screening of canine BPH.

### 2.2. Study 2: Development and Evaluation of a Web-Based Application for Canine BPH Severity Grading

#### 2.2.1. Study Population and Clinical Classification

Ninety intact male dogs owned by clients and undergoing evaluation for benign prostatic hyperplasia (BPH) were randomly selected from all cases of canine BPH evaluated by Observer 1 during 2025. BPH was diagnosed based on clinical signs, physical examination, ultrasonographic examination of the reproductive tract, and prostatic cytology. All dogs were examined by the same veterinarian (Observer 1), who had clinical experience in small-animal reproduction and the assessment of canine prostatic disorders. For each case, Observer 1 collected the clinical history, performed the physical and diagnostic examinations, and completed a standardised clinical record.

Based on the clinical findings and the Zambelli’s Symptom Index score (ZSI) [10], which assessed urethral discharge, haematuria, dyschezia/tenesmus, and dysuria, dogs were assigned to one of four BPH severity grades: 0 (asymptomatic BPH, with no clinical signs attributable to BPH), 1 (mild BPH), 2 (moderate BPH), or 3 (severe BPH). The classification established by Observer 1 served as the reference for subsequent analyses and was named “paper-based ZSI”.

#### 2.2.2. Clinical Record Review and Online Questionnaire Assessment

To assess the reproducibility of BPH grading among veterinarians, three additional clinicians (Observers 2–3–4), all with clinical experience in small-animal reproduction and in assessing canine prostatic disorders, independently reviewed a subset of clinical records generated by Observer 1. The 90 clinical records were randomly allocated among Observers 2–3–4, with 30 records assigned to each observer. The allocation was performed entirely at random and was independent of the BPH severity grade assigned by Observer 1, which was not disclosed to the observers.

The clinical records contained all information available at the initial examination, including signalment, clinical signs, and diagnostic findings. Based solely on the information reported in the records, each observer independently interpreted the case and completed an online questionnaire to assign a BPH severity grade (0 asymptomatic BPH, 1 mild BPH, 2 moderate BPH, or 3 severe BPH) to each patient evaluated. The authors retrieved this date from the server during the second stage, and this classification was called “app-based ZSI”.

**Figure 1:**
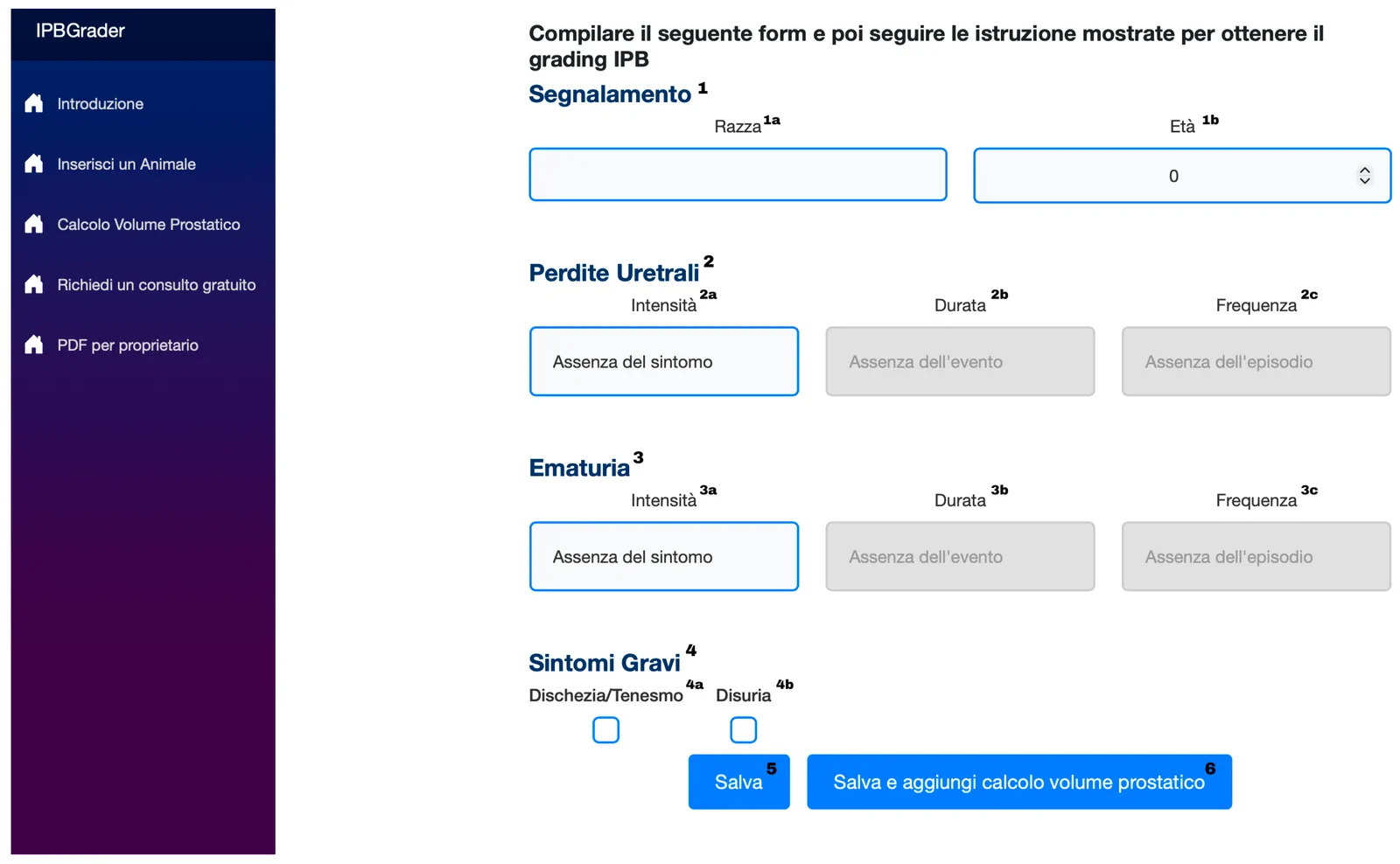
Representative view of the IPB Index web application showing the standardised electronic form for recording patient signalment and clinical manifestations, including urethral discharge, haematuria, dyschezia/tenesmus, and dysuria, to assign a benign prostatic hyperplasia severity grade. The platform interface is currently available in Italian. For clarity, the main interface elements shown in the screenshots are translated in the accompanying legend: 1—Signalment, 1a—Breed, 1b—Age; 2—Urethral discharge, 2a—Intensity, 2b—Duration, 2c—Frequency; 3—Haematuria, 3a—Intensity, 3b—Duration, 3c—Frequency; 4—Severe symptom, 4a—Dyschezia/tenesmus, 4b—Dysuria; 5—Save; 6—Save and add prostate volume calculation.

The questionnaire was available via the dedicated web platform (www.IPBIndex.net, accessed on 25 May 2026), developed as a web application using the .NET 8 framework with a C# backend and a Blazor-based frontend. The platform consists of a standardised electronic form designed to assign a BPH severity grade. The form comprises two main sections: patient signalment (including breed and age) and clinical manifestations, including urethral discharge, haematuria, dyschezia/tenesmus, and dysuria (Figure 1). Urethral discharge and haematuria are characterised by intensity, duration, and frequency via predefined dropdown menus to ensure standardised data collection. Based on the submitted clinical data, the platform assigns a BPH severity grade and generates a unique identification code for each case, allowing subsequent review by the Small Animal Reproduction Unit, Department of Veterinary Medical Sciences, University of Bologna. An optional section allows users to enter ultrasonographic prostatic measurements, enabling automatic calculation of expected and actual prostatic volumes according to previously published methods [13,14,15]. Detailed instructions for using the web platform are provided in Appendix A.

For each case, Observers 2, 3, and 4 assigned a BPH severity grade via the online questionnaire, using the same classification system as Observer 1 (asymptomatic, mild, moderate, or severe BPH). No discussion among observers was permitted during the evaluation.

#### 2.2.3. Comparison of Classifications

The classifications assigned by Observers 2–3–4 through the online application (app-based ZSI) were compared with the reference classification established by Observer 1 (paper-based ZSI). Similarly, the mean scores for urethral discharge, haematuria, and “other clinical signs” (dyschezia and tenesmus) assigned by Observer 1 were compared with those obtained by Observers 2–4 using the online application. Agreement between the original grading (Observer 1) and the classifications assigned by Observers 2–3–4 through clinical record review and use of the online application was assessed to evaluate the consistency and reproducibility of BPH grading.

Statistical analyses were performed in R version 4.5.3. Agreement between app-based and paper-based ZSI scores was assessed in 90 paired observations using exact percentage agreement and Cohen’s weighted kappa with quadratic weights, calculated with the psych package. Ninety-five percent confidence intervals were reported for the main agreement estimates, with bootstrap estimation used for weighted kappa. Agreement was also evaluated separately for the individual ordinal components contributing to the final ZSI grade. Spearman’s rank correlation coefficient was calculated as a complementary measure of association between app-based and paper-based ZSI scores.

## 3. Results

### 3.1. Study 1

The questionnaire was administered live and anonymously during the webinar to approximately 300 attendees. The number of respondents ranged from 148 to 216 (49–72%), depending on the questionnaire item, as respondents were not required to answer every question. All findings from Study 1 are therefore based exclusively on responses from webinar participants. Percentages were calculated based on the number of responses for each question.

Regarding screening practices, half of respondents routinely screened intact dogs, while the other half screened both intact and neutered dogs for prostatic diseases. Furthermore, 63% reported recommending starting screening at every annual check-up from age 5 onwards. Digital rectal examination (DRE) combined with ultrasonography was the preferred screening method. Detailed answers are reported in Table 1.

Concerning the diagnostic work-up, the majority of respondents reported serosanguineous, bloody, or purulent urethral discharge, haematuria, constipation/tenesmus and/or dysuria, and urinary retention/urinary obstruction as the most common symptoms associated with prostate diseases, and recommend ultrasonography as the initial diagnostic step. Fine-needle aspiration (FNA) of the prostatic parenchyma and any cysts, if present, collection of seminal fluid, and urine collection by cystocentesis were indicated as the principal methods for obtaining prostatic samples to investigate prostatic disease. Detailed answers are reported in Table 2.

**Figure 2:**
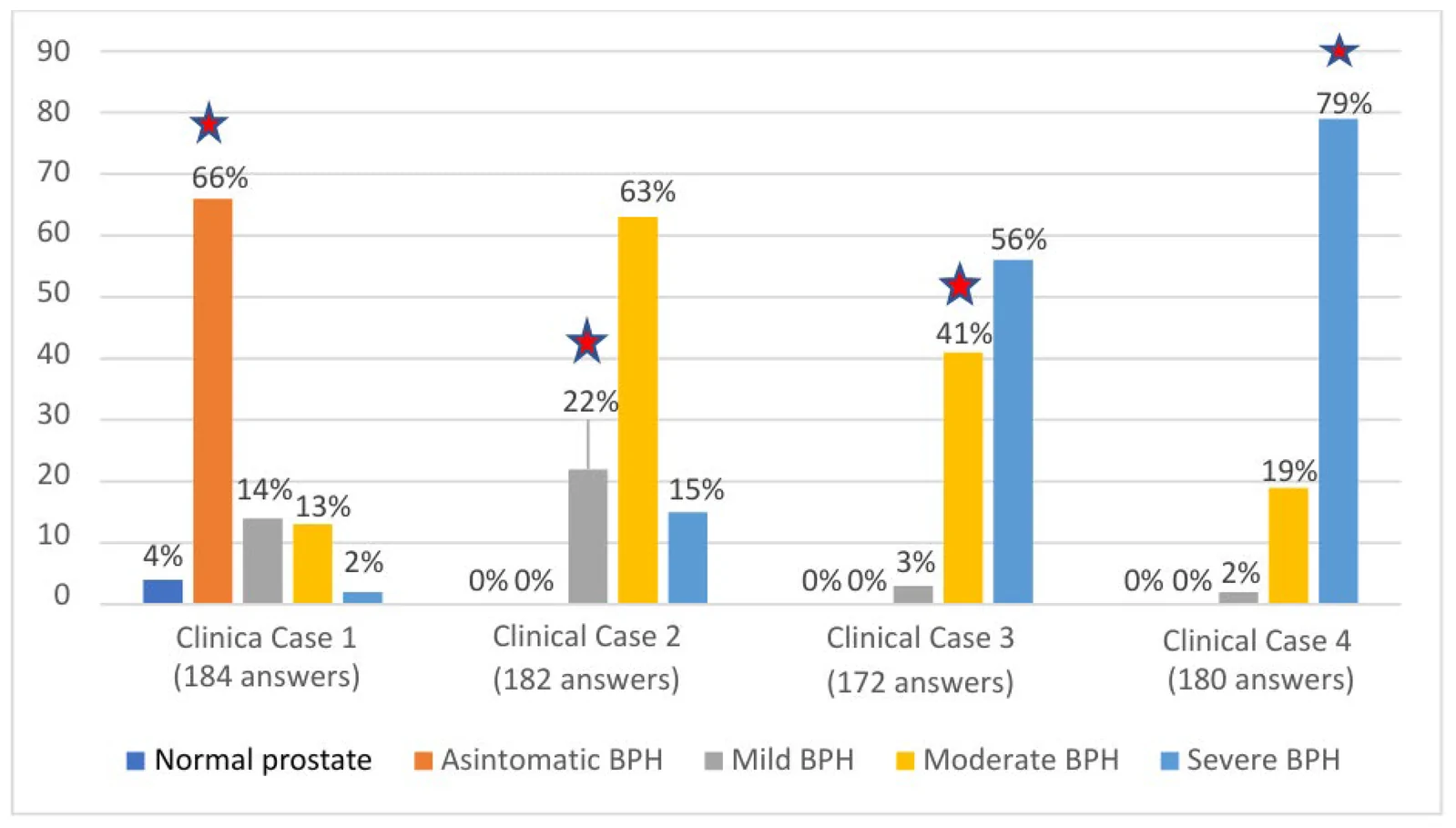
Distribution of respondents’ assessments of BPH severity based on the information provided for each clinical case. The asterisk indicates the BPH severity grade assigned by the questionnaire developers using the ZSI for the corresponding case.

Practitioners’ assessment of BPH severity was evaluated using four clinical cases followed by a survey question (Figure S1–S4). For each clinical case, the signalment, clinical signs, CPSE concentration, prostate dimensions and volume (expected and measured), and ultrasonographic images were provided. This data reflects the clinical information participants had when responding to the related questionnaire questions. The distribution of responses across the four clinical cases is presented in Figure 2. The survey question, administered after the clinical cases, assessed the primary factor respondents considered when evaluating BPH severity. The majority of respondents reported relying primarily on clinical signs (39%) or on a combination of CPSE concentration and ultrasonographic findings (37%). Detailed responses are presented in Table 3.

**Figure 3:**
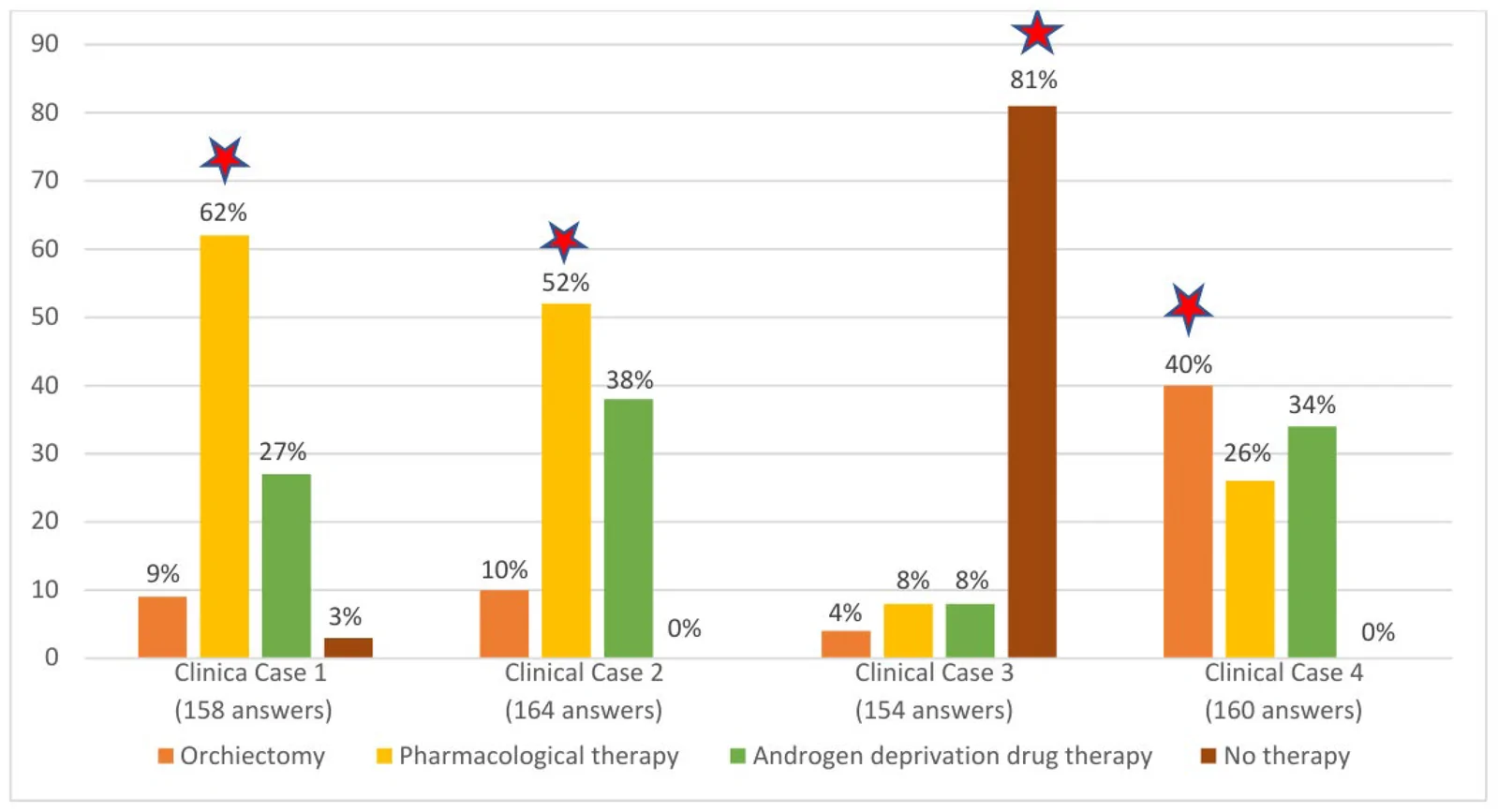
Distribution of respondents’ therapeutic decisions based on the information provided for each clinical case. The asterisk indicates the therapeutic approach suggested by the questionnaire developers based on the ZSI score assigned to the corresponding case.

Regarding BPH management, most respondents reported that they would perform additional diagnostic testing when an enlarged prostate is detected on DRE in an intact dog. For treatment, 34% of respondents indicated that they would prescribe osaterone. Detailed responses are presented in Table 4. Additionally, four clinical cases were included in the questionnaire to assess respondents’ approach to BPH management (Figure S5–S8). For each case, the signalment, clinical signs, CPSE concentration, prostate dimensions and volume (expected and measured), and ultrasonographic images were provided. These data represented the clinical information available to participants when answering the corresponding questionnaire questions. The distribution of responses for each clinical case is shown in Figure 3.

### 3.2. Study 2

**Figure 4:**
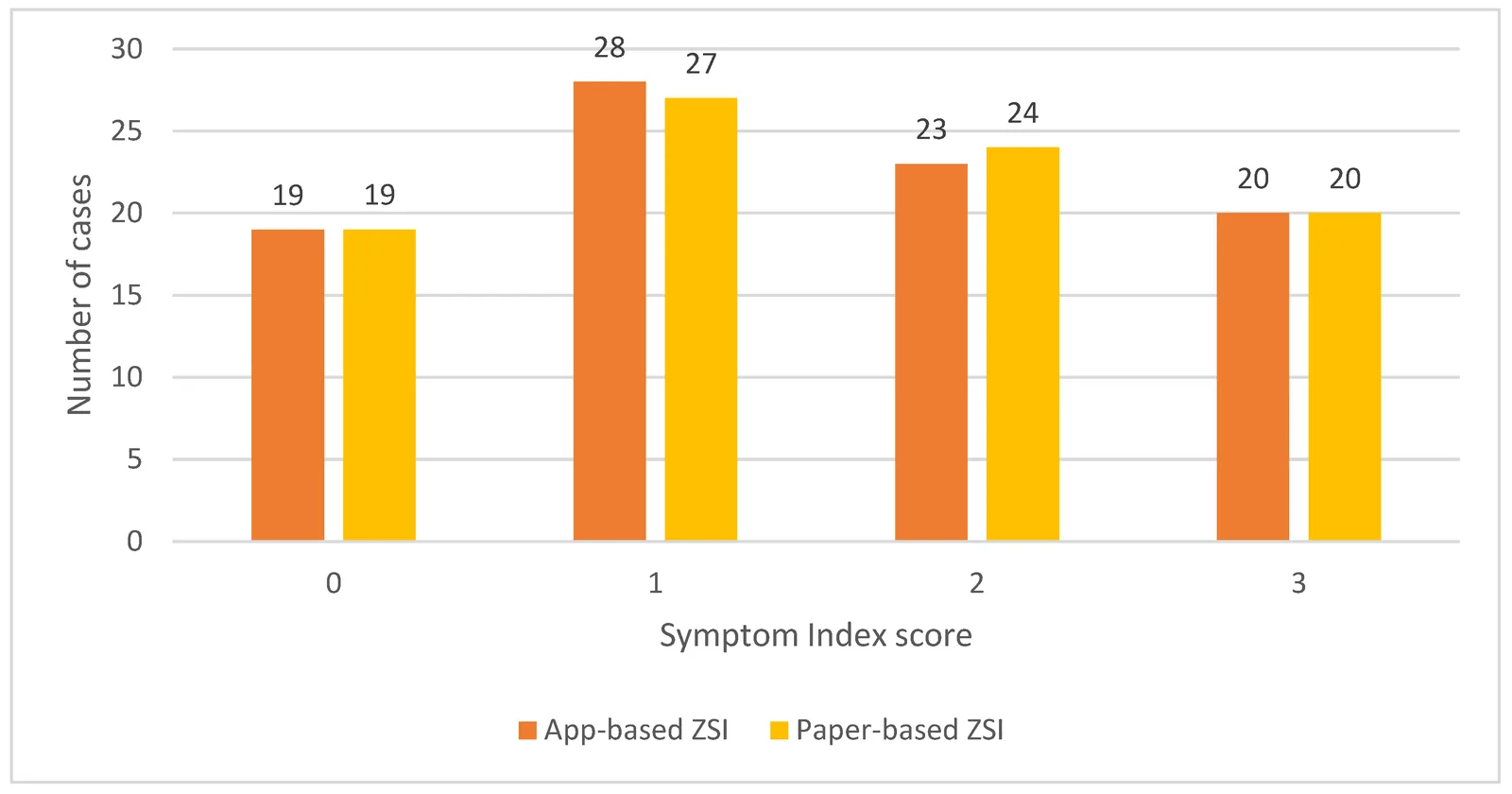
Distribution of Symptom Index scores across the two methods.

Agreement between the app-based and paper-based Symptom Index scores was assessed using 90 paired observations. The distribution of Symptom Index scores was comparable across the two methods, with identical frequencies for scores 0 and 3 and differences of only one observation for scores 1 and 2, as shown in Figure 4.

Overall, 87 of 90 paired observations were concordant, corresponding to exact agreement of 96.7% (95% CI, 90.6–99.3%). The weighted Kappa was 0.985 (95% bootstrap CI, 0.964–1.000), indicating almost perfect agreement between the two scoring methods. Spearman’s rank correlation coefficient was 0.983. Detailed data are reported in Table 5.

Only three discordant cases were identified, and all differed by a single Symptom Index category; no discrepancies involving two or more categories were observed. The disagreements were mainly attributable to differences in the component scores for Leak Average and Ematuria Average. Agreement was consistently high for the individual SI components. Exact agreement was 95.6% (weighted κ = 0.965) for Leak Average, 96.7% (weighted κ = 0.856) for Ematuria Average, and 100% (weighted κ = 1.000) for the Other component, as shown in Table 6.

The agreement between app-based and paper-based Symptom Index scores was consistently high across all operators, with exact agreement ranging from 93.3% to 100% and weighted Kappa values from 0.961 to 1.000. Data are shown in Table 7.

Exploratory analyses found no significant correlation between Symptom Index score and either measured or expected prostate volume, irrespective of the scoring method (all *p* > 0.05).

## 4. Discussion

### 4.1. Study 1

This survey provides an overview of current approaches to screening, diagnosis, severity assessment, and management of canine benign prostatic hyperplasia among veterinarians attending an educational webinar on male canine reproductive medicine. Overall, the findings revealed broad agreement on certain aspects of the diagnostic approach, particularly the use of ultrasonography and the need for further investigation of prostatic enlargement. Conversely, substantial variability was observed in the recognition of clinical signs, the assessment of disease severity, and the selection of therapeutic strategies. These findings suggest that, despite the high prevalence and clinical relevance of BPH in intact male dogs, its assessment and management remain incompletely standardised in clinical practice.

Participation in the voluntary, anonymous survey varied across questionnaire items, indicating that not all webinar attendees completed every question. Nevertheless, the number of responses was considered sufficient to provide meaningful descriptive information on the opinions and reported practices of participating veterinarians. Potential non-response and selection biases should, however, be acknowledged. Although the webinar focused on canine reproduction, attendees were not necessarily reproductive specialists or practitioners with a specific interest in this area. The webinar was part of a series addressing different topics of current interest in veterinary medicine; therefore, its audience was likely to include veterinarians with diverse professional interests and backgrounds. Consequently, the findings should not be interpreted as representative only of the practices and opinions of reproductive specialists or veterinarians with a particular interest in canine reproduction. Rather, they provide descriptive insights into the opinions and reported practices of veterinarians who participated in the webinar, and caution should be taken when extrapolating these findings to the broader veterinary community.

Several measures were adopted to limit response bias. In particular, all questions and clinical cases were presented before the corresponding topics were explained or discussed during the webinar. Furthermore, the order of the questions was predetermined to minimise the possibility that earlier educational content would influence subsequent responses. Nonetheless, in some cases, the single-answer multiple-choice format may have influenced respondents’ choices and might not have fully captured the complexity of their clinical reasoning.

#### 4.1.1. Screening Practices

Half of respondents screened only intact dogs for prostatic diseases, whereas the other half screened both intact and neutered dogs. The question addressed screening for prostatic diseases in general and was not limited to BPH. Although BPH is an androgen-dependent disorder that primarily affects sexually intact male dogs, screening both intact and neutered dogs is relevant because prostatic disorders other than BPH and metaplasia can occur in both populations. In particular, prostatic neoplasia is not prevented by castration and may therefore warrant consideration when screening for prostatic diseases in neutered dogs [16,17].

Most respondents recommended initiating routine screening at 5 years of age. However, histological changes consistent with BPH may develop considerably earlier. In a study of beagles, histological evidence of BPH was detected in 16% of dogs by 2 years of age and in approximately 50% by 4.1–5 years of age [18]. Although histological hyperplasia does not necessarily correspond to clinically relevant disease, its early occurrence supports considering prostatic assessment before 5 years of age, particularly in breeding dogs or in individuals presenting with subtle reproductive, urinary, or gastrointestinal abnormalities.

The combination of DRE and ultrasonography was the most commonly selected screening approach. These methods provide complementary information: DRE allows an initial assessment of prostatic size, symmetry, position, consistency, mobility, and discomfort, whereas ultrasonography enables a more detailed evaluation of prostatic dimensions, volume, parenchymal echotexture, focal lesions, cystic changes, and surrounding structures [16,17,19]. In addition, DRE alone has limited sensitivity for detecting subclinical prostatic disease. In a post-mortem study of 500 male dogs, DRE showed a sensitivity of 53% and a specificity of 75%, supporting its use as part of, rather than as a substitute for, a multimodal screening approach [20]. Using both DRE and ultrasonography is probably ideal for prostatic cancer screening in neutered dogs, but for BPH screening in intact dogs, combining DRE with serum canine prostate-specific esterase (CPSE) measurement may be a practical preliminary approach, particularly when ultrasonography is not immediately available. Serum CPSE concentration is positively associated with prostate size and has shown good diagnostic performance in differentiating dogs with cytologically confirmed BPH from unaffected middle-aged intact dogs [21,22]. However, CPSE is a prostate-specific but not disease-specific biomarker, as increased concentrations may also occur in prostatitis and prostatic neoplasia [23]. Therefore, neither CPSE measurement nor ultrasonography should be considered independently diagnostic of BPH. Rather, screening results should be interpreted in conjunction with the dog’s clinical signs, age, reproductive status, physical examination findings, and, when indicated, additional diagnostic tests.

#### 4.1.2. Diagnostic Work-Up

Responses regarding the clinical manifestations of prostatic disease were heterogeneous. Urethral discharge and haematuria were the most frequently selected individual signs, whereas almost half of respondents indicated that all the listed signs were generally present. This may suggest difficulty in distinguishing signs commonly associated with BPH from those more suggestive of other prostatic disorders.

Uncomplicated BPH is often subclinical. When present, the most commonly reported signs include intermittent serosanguineous urethral discharge, haematuria, haematospermia, tenesmus, and changes in faecal shape. Conversely, prostatic pain, systemic illness, purulent discharge, marked dysuria, and urinary obstruction may raise greater suspicion of concurrent prostatitis, abscessation, cystic disease, or neoplasia [16,17,19]. The frequent selection of all the listed signs may therefore reflect a tendency to consider prostatic diseases as a single clinical entity rather than as a heterogeneous group of conditions with partially overlapping presentations. This underlines the importance of a structured diagnostic approach and targeted continuing education that emphasises the distinction between uncomplicated BPH and other prostatic disorders.

Most respondents selected ultrasonography as the initial diagnostic procedure for an intact dog with prostatic enlargement. This aligns with the central role of ultrasonography in assessing prostatic size, volume, parenchymal homogeneity, cystic lesions, and surrounding tissues [16,19]. However, ultrasonographic abnormalities are not always specific, and BPH, prostatitis, cystic disease, and neoplasia may have overlapping appearances. Ultrasonography should therefore be considered an essential component of the initial diagnostic evaluation, but not a definitive stand-alone test [17,19].

Additional investigations should be selected according to the clinical presentation and ultrasonographic findings. Cytological examination may be required when neoplasia, prostatitis, or atypical lesions are suspected, whereas bacteriological culture is indicated when infection is likely [17,19]. Similarly, aspiration and biochemical or cytological evaluation of cystic fluid may be useful in selected cases. The risks and diagnostic benefits of prostatic sampling should always be carefully assessed, particularly when abscessation or neoplasia is suspected.

Responses on sampling methods also showed substantial variability. The most commonly selected approach was to choose between prostatic massage, FNA, and semen collection based on the suspected diagnosis. This finding appropriately reflects that no single sampling method is optimal for every prostatic disorder. Prostatic massage and semen collection may yield material from the prostate but can be contaminated by the lower urinary or genital tract. FNA can provide direct cytological samples from focal or diffuse lesions but should preferably be performed under ultrasonographic guidance, with careful consideration of potential complications. Urine collected by cystocentesis is particularly useful for bacteriological examination of the urinary tract, although a negative result does not necessarily exclude localised prostatic infection [17,19]. The selection of the sampling procedure should therefore be guided by the suspected disease, the location and nature of the lesion, and the type of laboratory analysis required.

#### 4.1.3. Assessment of BPH Severity

Only 39% of respondents identified clinical signs as the primary factor in assessing BPH severity, whereas 37% selected the combination of serum CPSE concentration and ultrasonographic findings. The distribution of severity grades assigned to clinical cases also showed considerable variability among respondents. Agreement with the reference classification based on the Symptom Index was more evident in some cases than in others, suggesting that participants did not apply consistent criteria across clinical presentations.

These findings highlight the absence of a universally adopted method for grading the clinical severity of canine BPH. Prostatic size, ultrasonographic abnormalities, and serum CPSE concentrations can contribute to the detection and characterisation of BPH, but they do not necessarily reflect the severity of its clinical consequences. CPSE concentration has been associated with prostate size [22,23,24] and, in a recent study, with clinical severity and ultrasonographic alterations [12]. Nevertheless, structural or biomarker abnormalities do not necessarily correspond directly to the severity of clinical disease. Dogs with considerable prostatic enlargement may remain asymptomatic, whereas clinically relevant signs may occur in dogs with less marked structural changes [1,22,24].

At the same time, clinical signs alone may not adequately distinguish uncomplicated BPH from concurrent prostatic disease. An integrated assessment combining history, symptoms, clinical examination, DRE, serum CPSE concentration, and ultrasonographic findings is therefore necessary to establish the diagnosis. Once other clinically relevant prostatic conditions have been excluded, a symptom-based index may offer a more reproducible method for grading the clinical expression of BPH and for assessing the need for treatment.

#### 4.1.4. Management of BPH

Most respondents indicated they would perform further diagnostic investigations when prostatic enlargement was detected by DRE in an intact dog. This is appropriate approach, as enlargement is not specific to BPH and may also be associated with prostatitis, cysts, abscessation, or neoplasia [9,10,12]. Neither DRE findings nor an increased CPSE concentration, in isolation, should be considered sufficient to establish a definitive diagnosis. Additional investigations help identify alternative or concurrent disorders and support the selection of an appropriate therapeutic strategy.

Osaterone acetate was the most commonly selected individual pharmacological treatment, followed by deslorelin and finasteride. The preference for osaterone acetate may reflect its specific authorisation in Europe for treating canine BPH, its relatively rapid clinical effect, and the preservation of fertility-related functions after a temporary treatment period [11,25]. Deslorelin may be selected when longer-term suppression of androgen-dependent prostatic activity and temporary infertility are acceptable or desired [11,26]. Finasteride was selected less frequently. Its limited use may reflect its off-label status in veterinary medicine in some jurisdictions, as well as differences in availability and clinicians’ familiarity with the drug. Nevertheless, finasteride has been shown to reduce prostatic volume and serum dihydrotestosterone concentrations without adversely affecting sperm quality or serum testosterone concentration, although semen volume may decrease [27].

Therapeutic decisions for the clinical cases remained variable, although the Symptom Index-based approach was selected by the largest proportion of participants in most cases. This variability aligns with the observed differences in severity grading and suggests that uncertainty in classifying BPH may directly influence treatment decisions. In particular, clinicians who rely primarily on prostate size, ultrasonographic appearance, or CPSE concentration may be more inclined to initiate treatment in asymptomatic dogs, whereas those using a symptom-based approach may reserve treatment for dogs with clinically relevant disease.

The decision to treat should take into account the severity of clinical signs, diagnostic findings, intended breeding use, the need to preserve fertility, the presence of concurrent disease, and owner preferences. Asymptomatic prostatic enlargement does not necessarily require immediate pharmacological treatment, although periodic monitoring may be appropriate. Although orchiectomy has traditionally been considered the treatment of choice for androgen-dependent BPH in dogs not intended for breeding [16,17,19], current guidelines support a more individualised approach, in which medical therapy is a valid alternative, particularly when fertility preservation or temporary suppression of prostatic activity is desired [6].

The present study’s findings reveal both areas of agreement and substantial heterogeneity in veterinarians’ approaches to canine BPH. Most respondents recognised the importance of ultrasonography and further diagnostic evaluation of prostatic enlargement. However, variability in interpreting clinical signs, assessing severity, and selecting treatment indicates that a standardised clinical pathway for BPH has not yet been universally adopted.

An integrated diagnostic approach is required to distinguish BPH from other prostatic diseases, whereas the clinical severity of confirmed BPH can be assessed more consistently using a validated symptom-based scoring system. Wider adoption of standardised diagnostic and grading criteria could improve communication among clinicians, support more consistent therapeutic decisions, and facilitate comparisons of outcomes across clinical studies. Further research should evaluate the reproducibility, clinical validity, and responsiveness to treatment of symptom-based BPH grading systems in larger and more representative veterinary populations.

### 4.2. Study 2

The present study demonstrated a very high level of agreement between app-based and paper-based application of the Symptom Index for grading the clinical severity of canine benign prostatic hyperplasia. Exact agreement was observed in 96.7% of the 90 paired assessments, and the quadratically weighted kappa of 0.985 indicated almost perfect agreement. Taken together, these findings suggest that implementing the Symptom Index in an online application does not materially alter the final severity classification and may provide a reliable alternative to conventional paper-based scoring.

The high agreement observed in this study is particularly relevant, as Zambelli’s Symptom Index was originally developed to standardise the assessment of canine BPH severity based on clinical signs reported by owners or observed by veterinarians. The original model correctly classified 97.3% of cases, supporting its potential usefulness for standardising clinical assessment and guiding treatment decisions [10]. The present findings extend these observations by showing that the scoring system can be transferred to a digital format without compromising its classification performance. Digital implementation may facilitate routine use by providing a structured approach to data entry, reducing errors in score calculation, and enabling standardised recording and retrieval of clinical information.

Only three discordant classifications were recorded, and all differed by a single severity category. The absence of disagreements spanning two or more categories indicates that the observed discrepancies were minor and unlikely to result from fundamentally different interpretations of the dogs’ clinical status. Nevertheless, even a one-category difference could influence clinical decisions, particularly when a patient is classified close to a threshold separating asymptomatic from mild disease or moderate from severe disease. Cases with scores close to category boundaries should therefore be interpreted alongside the complete clinical and diagnostic assessment.

The discordant classifications were mainly related to the Leak Average and Hematuria Average components. Although both variables showed high exact agreement, the slightly lower kappa for Hematuria Average compared with Leak Average may indicate greater variability in the interpretation or recording of this clinical sign. This difference should be interpreted cautiously, as agreement remained high for both components.

Variability may partly reflect the fact that information on the amount, frequency, and duration of urethral leakage or haematuria is generally based on owner observations and may be reported using imprecise or non-standardised descriptions. Importantly, urethral discharge and haematuria are distinct clinical signs, but owners may have difficulty distinguishing between them, particularly when blood is observed independently of urination or when a haemorrhagic urethral discharge is mistaken for haematuria. Therefore, it is important that veterinarians clearly explain the distinction between these two clinical signs and specifically investigate their characteristics during history taking to obtain accurate and clinically relevant information. Providing clearer definitions, explanatory notes, or illustrative examples within the application may further support veterinarians in guiding owners and standardising data entry.

Agreement was consistently high across all three independent operators; these results suggest that app-based classification remained robust regardless of the clinician conducting the assessment.

The standardised clinical records probably contributed to the high level of agreement by ensuring that Observers 2–3–4 had access to the same clinical and diagnostic variables collected by Observer 1. However, the application-based assessment itself is designed to standardise both data entry and interpretation. Users are guided by specific instructions when entering predefined variables. Consequently, when the same input data are entered correctly, the application generates the same final classification, irrespective of the setting in which the information was collected or the level of additional detail included in the clinical record. The reproducibility of the final classification therefore does not depend on subjective weighting of the available information by individual users. Any potential variability would arise primarily from differences or inaccuracies in the initial clinical assessment, measurement, or transcription of the required variables, rather than from the application’s interpretation of those data.

Observer 1 examined the dogs directly, whereas Observers 2–3–4 based their assessments on information documented in the clinical records. Therefore, this comparison did not constitute a conventional interobserver study in which multiple clinicians independently examine the same patients. Instead, it evaluated whether the standardised clinical and diagnostic information collected during the original examination could be correctly entered into the application and reproduce the reference classification assigned using the same standardised system. The high level of agreement therefore supports the reproducibility and internal consistency of the application-based classification when the required input variables are correctly recorded and entered. However, it does not assess agreement among clinicians in the acquisition or interpretation of the original clinical findings. Future studies involving independent examinations of the same dogs by multiple clinicians would be useful to determine the extent to which variability in clinical data acquisition may influence the resulting application-based classification. A principal limitation of this study is that observers evaluated different sets of 30 cases. This design precluded direct comparisons among observers using the same clinical records. Further studies conducted in general practice settings, with multiple observers assessing the same cases, would allow a more comprehensive evaluation of interobserver agreement and may help identify the clinical variables most susceptible to subjective interpretation.

In conclusion, the online application provided BPH severity classifications that were highly consistent with those obtained using the paper-based ZSI. Disagreements were uncommon, confined to adjacent severity categories, and mainly related to the urethral leakage and haematuria components. These findings support the use of this digital tool for the standardised recording and classification of clinical BPH severity.

Note: at present, the online platform is not optimised for mobile devices and does not provide a fully optimised English translation; therefore, the use of the browser’s automatic translation feature is recommended. For proper and comprehensive use of the available functionalities, access to the website via the Google Chrome browser is advised.

## 5. Conclusions

This study highlights the need for greater standardisation in the clinical assessment of canine benign prostatic hyperplasia. The survey revealed substantial variability among participating veterinarians in recognising clinical signs, assessing BPH severity, and selecting therapeutic strategies, despite broader agreement on the role of ultrasonography and the need for further diagnostic investigation of prostatic enlargement. These findings suggest that the clinical severity of canine BPH is not currently assessed using uniform criteria and support the use of a structured, symptom-based approach after other clinically relevant prostatic disorders have been excluded.

The web-based implementation of the Zambelli Symptom Index showed very high agreement with the reference paper-based classification, with discrepancies confined to a small number of cases and to adjacent severity categories. These findings demonstrate that the ZSI can be transferred to a digital platform without materially altering the resulting BPH severity classification, and support its use as a standardised tool for recording and grading the clinical expression of canine BPH.

The proposed web-based approach may facilitate consistent use of the Symptom Index in clinical practice and research. However, further studies involving multiple clinicians independently examining the same dogs in general practice settings are warranted to assess interobserver variability in clinical data collection and to evaluate the digital scoring system’s clinical validity, responsiveness to treatment, and broader applicability.

## Figures and Tables

**Table 1:** Answers to questions about screening practices.

***At what age do you recommend starting screening, and how often do you recommend it? (168 answers)***	
At every annual check-up from puberty onward	5%
At every annual check-up from 3 years of age onward	13%
At every annual check-up from 5 years of age onward	63%
At every annual check-up only in senior dogs	19%
***Which methods do you routinely use to screen dogs for prostatic diseases? (164 answers)***	
I do not perform screening.	9%
DRE	4%
DRE and CPSE testing	4%
CPSE testing	2%
Ultrasonography	10%
CPSE testing and ultrasonography	18%
DRE and ultrasonography	54%

**Table 2:** Answers to questions about diagnostic work-up.

***What is the most common symptom you typically observe in patients with prostate diseases? (216 answers)***	
Constipation/tenesmus (ribbon-like stools)	6%
Serosanguineous, bloody, or purulent urethral discharge	30%
Hematuria	18%
Dysuria, urinary retention/urinary obstruction	1%
All of the above symptoms are usually present in all cases	45%
***Which of the following do you recommend as the initial diagnostic work-up for an intact dog with an enlarged prostate? (184 answers)***	
Ultrasonography	86%
Cytological examination and bacteriological culture	0%
Biochemical analysis of cyst fluid (if a cyst is present)	0%
All of the above	14%
***When investigating a suspected prostatic disease, which method(s) do you use to collect prostatic samples? (148 answers)***	
Method 1: Prostatic massage	14%
Method 2: Fine-needle aspiration (FNA) of the prostatic parenchyma and any cysts, if present	9%
Method 3: Collection of seminal fluid	1%
Method 4: Urine collection by cystocentesis	5%
Method 5: Voided urine sample	5%
Methods 1, 2, and 3, depending on the suspected diagnosis	42%
Methods 1, 3, and 4, as they are associated with lower risk, particularly when infection or neoplasia is suspected	15%
Methods 4 and 5, as they are non-invasive and still diagnostically useful	8%

**Table 3:** Answers to questions about the primary factors considered when assessing BPH severity.

***Which of the following factors do you primarily consider when assessing the severity of BPH? (180 answers)***	
Ultrasonographic findings (parenchymal appearance, presence of cysts, and prostate size)	23%
Clinical signs	39%
CPSE	0%
Combination of CPSE and ultrasonographic findings	37%
Combination of DRE and CPSE	1%

**Table 4:** Answers to questions about the management of BPH.

***How do you manage an intact dog with an enlarged prostate detected on digital rectal examination? (152 answers)***	
I perform further diagnostic investigations.	93%
I recommend orchiectomy (castration).	0%
I recommend medical treatment.	0%
If the dog is asymptomatic, I do not recommend any intervention.	7%
***Which of the following medications do you currently use, or have you used in the past, to treat benign prostatic hyperplasia (BPH)? (196 answers)***	
Finasteride	7%
Osaterone	34%
Deslorelin	10%
Cyproterone/Flutamide	0%
Finasteride, Osaterone, Deslorelin, Cyproterone/flutamide	4%
Finasteride, Osaterone, Deslorelin	15%
**Finasteride, Deslorelin**	9%
Osaterone, Deslorelin	21%

**Table 5:** Agreement between app-based and paper-based ZSI. Exact agreement, weighted Kappa with quadratic weights, and the corresponding 95% confidence intervals are reported for the overall Symptom Index score.

Indicator	Result
Concordant	87/90
Exact agreement	96.7% (95% CI)
95% CI of the agreement	90.6–99.3%
Weighted quadratic Kappa	0.985
Bootstrap 95% CI of the quadratic Kappa	0.964–1.000
Spearman ρ	0.983

**Table 6:** Agreement between app-based and paper-based scoring for the individual Symptom Index components. Exact agreement and weighted Kappa with quadratic weights are reported for each component.

Symptom Index Category	Concordant	Agreement	Weighted Quadratic Kappa
Leak Average	86/90	95.6%	0.965
Ematuria Average	87/90	96.7%	0.856
Other	90/90	100.0%	1.000

**Table 7:** Agreement between app-based and paper-based ZSI scores, stratified by operator. Exact agreement with corresponding 95% confidence intervals and Cohen’s weighted kappa with quadratic weights are reported for each operator.

Operator	Number of Cases	Concordant	Agreement	IC 95%	Weighted Quadratic Kappa
A	30	29/30	96.7%	82.8–99.9%	0.987
B	30	30/30	100.0%	88.4–100.0%	1.000
C	30	28/30	93.3%	77.9–99.2%	0.961

**Figure A1:**
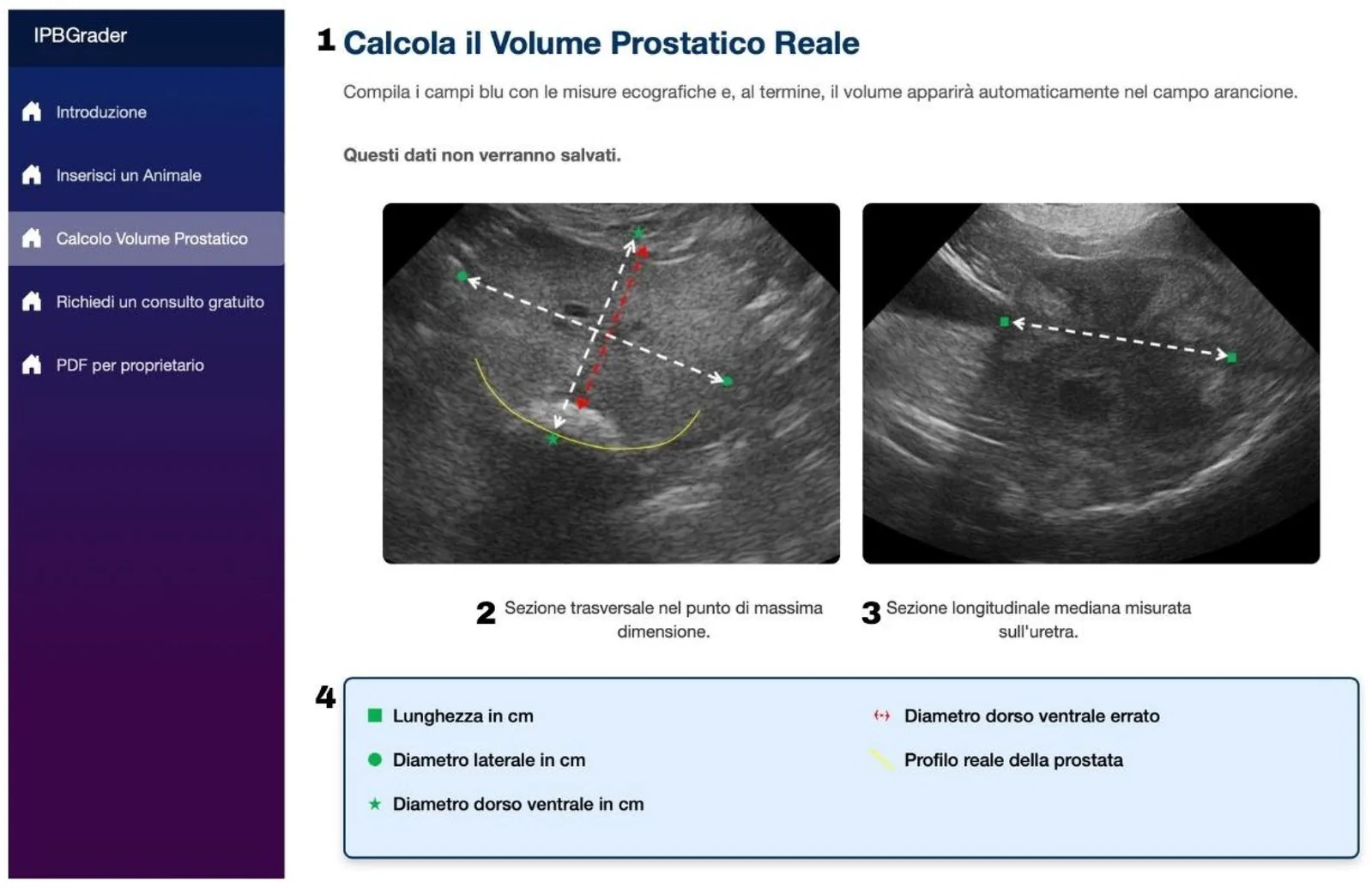
Representative view of the IPB Index web application, showing the two reference images provided to help users obtain measurements at the indicated anatomical reference points. The platform interface is currently available in Italian. For clarity, the main interface elements shown in the screenshots are translated in the accompanying legend: 1—Calculate the actual prostate volume; 2—Transversal section at the point of maximum dimension; 3—Median longitudinal section measured in the urethra; 4—The blue box provides the legend for the measurements indicated in the figure. On the right side of the box: prostate length (cm), transverse diameter (cm), and dorsoventral diameter (cm). On the left side of the box: incorrect dorsoventral diameter (cm) and actual prostatic profile.

**Figure A2:**
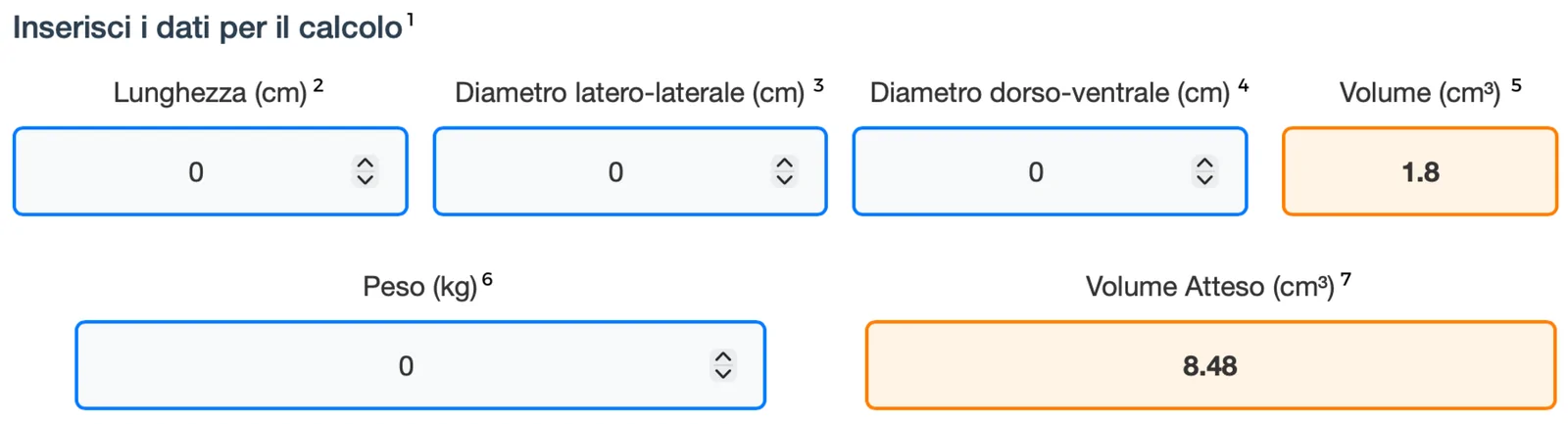
Representative view of the IPB Index web application, showing the calculation side for prostatic volume (Volume Atteso) and the actual prostatic volume (Volume). The platform interface is currently available in Italian. For clarity, the main interface elements shown in the screenshots are translated in the accompanying legend: 1-Enter the data for the calculation; 2-Length (cm); 3-Latero-lateral diameter (cm); 4-Dorso-ventral diameter (cm); 5-Actual volume (cm^3^); 6-Weight (kg); 7-Expected volume (cm^3^). Note that non-zero actual and expected volume values may be displayed even when zero is entered for the prostatic measurements, because the volume calculation equations include a constant term. This reflects the mathematical structure of the equations implemented on the platform and does not indicate a computational error.

**Figure A3:**
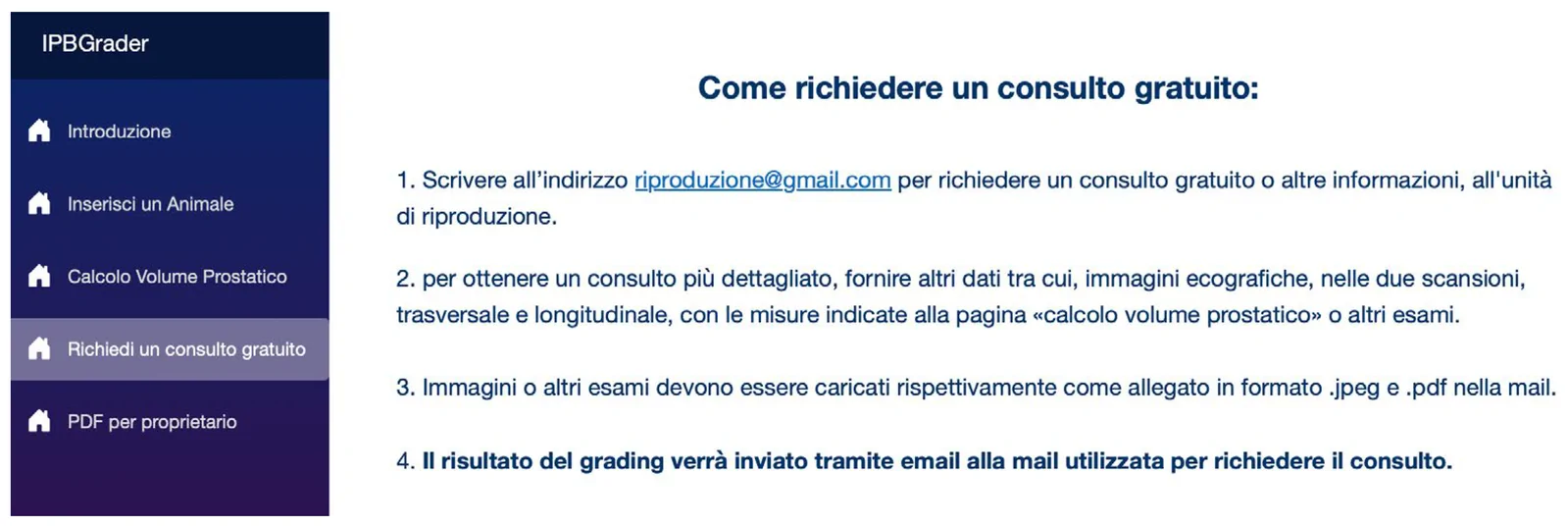
User interface of the “Request a Free Consultation” section, providing instructions for contacting the Small Animal Reproduction Unit, Department of Veterinary Medical Sciences, University of Bologna, to request a specialist consultation. The platform interface is currently available in Italian. For clarity, the instructions shown in the screenshot are translated into English as follows: 1—Contact the Small Animal Reproduction Unit at riproduzione@gmail.com to request a free consultation or further information; 2—For a more detailed consultation, provide additional information, including ultrasonographic images obtained in both transverse and longitudinal views, with the measurements indicated in the “Prostate Volume Calculation” section, or other relevant diagnostic examinations; 3—Images and other examination results should be attached to the email in .jpeg and .pdf formats, respectively; 4—The grading result will be sent by email to the address used to request the consultation.

## Data Availability

The original contributions presented in this study are included in the article. Further inquiries can be directed to the corresponding author.

## Supplementary Materials

The following supporting information can be downloaded at https://www.mdpi.com/article/10.3390/ani16182833/s1. A total of eight clinical scenarios were presented to the webinar participants to assess their ability to evaluate BPH severity and make therapeutic decisions. Figures S1–S4 show the original slides related to the clinical cases used to assess respondents’ ability to evaluate BPH severity, whereas Figures S5–S8 show the original slides related to the clinical cases used to assess respondents’ therapeutic decision-making.

## Appendix A. User Manual for the BPH Index Web Application

The website [www.IPBIndex.net, accessed on 25 May 2026] was developed as a web-based tool to assist veterinarians in assessing benign prostatic hyperplasia (BPH) in male dogs and to standardise grading of BPH severity.

### Appendix A.1. Landing Page and Clinical Data Entry

The website’s landing page displays a form for entering the information required to determine a BPH severity grade. The fields are divided into two sections: patient signalment, including breed and age, and clinical manifestations, including urethral discharge, haematuria, dyschezia/tenesmus, and dysuria (Figure 1).

Urethral discharge and haematuria must be characterised by intensity, duration, and frequency. To ensure standardised recording of these three parameters, each is entered via a predefined dropdown menu.

From the landing page, users can either save the completed form by clicking the “Save” button or proceed to the second section by selecting “Save and add prostate volume calculation”.

Once the “Save” button is clicked at the bottom of the page, a screen displays instructions on how to obtain the BPH severity grade. The platform automatically calculates the BPH severity grade based on the information entered by the user; however, the grade is not displayed directly to the user.

Each submitted case is assigned a unique identification code. To obtain the corresponding BPH severity grade, the veterinarian must email the Small Animal Reproduction Unit, Department of Veterinary Medical Sciences, University of Bologna, and provide the case’s unique identification code. The Unit can then retrieve the case and provide the BPH severity grade calculated by the platform.

### Appendix A.2. Prostate Volume Calculation

By selecting “Save and add prostate volume calculation,” users can enter prostatic measurements. The required measurements include the transverse diameter at the point of maximum prostatic width and the median longitudinal diameter measured along the urethral axis. Two reference images are provided to help users obtain measurements at the indicated anatomical reference points (see Figure A1).

At the bottom of the page, users can calculate both the expected prostatic volume according to Sannamwong et al. [13] and the actual prostatic volume according to Blum et al. [14] as cited in Ruel et al. [15], as shown in Figure A2.

### Appendix A.3. Website Menu

The left-hand menu provides access to the following sections:

- “Introduction”;
- “Add an Animal”;
- “Prostate Volume Calculation”;
- “Request a Free Consultation”;
- “PDF for Owner”.

The “Introduction” section offers a brief overview of BPH and emphasises the importance of grading disease severity in dogs presenting with clinical signs.

The “Add an Animal” section redirects users to the landing page, where a new clinical case can be entered. Multiple cases can be submitted in sequence. After submitting a case, users can contact the Small Animal Reproduction Unit, Department of Veterinary Medical Sciences, University of Bologna, by email and provide the unique identification code for each case.

The “Prostate Volume Calculation” section allows users to calculate both actual prostatic volume [14,15] and expected prostatic volume [13] without saving the entered data.

The “Request a Free Consultation” section allows users to contact the Small Animal Reproduction Unit, Department of Veterinary Medical Sciences, University of Bologna, directly without completing the BPH assessment form (Figure A3). The page provides instructions for requesting a specialist consultation and indicates the information that should be included in the email, including the identification code of the submitted case, when applicable.

The “PDF for Owner” section provides a downloadable document in both Italian and English, offering a concise explanation of BPH for dog owners.

### Appendix A.4. Technical Information

At present, the platform is not optimised for mobile devices and does not provide a fully optimised English-language version. When necessary, users are therefore advised to use the browser’s automatic translation function. For optimal and comprehensive access to all available features, use of the website through the Google Chrome browser is recommended.

The database storing information collected through the platform can be queried and managed using SQL Server Management Studio. Submitted cases can be retrieved using the unique identification code, which allows the corresponding BPH severity grade to be assigned to each clinical case.

## References

[1] Ruetten H., Wehber M., Murphy M., Cole C., Sandhu S., Oakes S., Bjorling D., Waller K., Viviano K., Vezina C. (2021). A retrospective review of canine benign prostatic hyperplasia with and without prostatitis. Clin. Theriogenol..

[2] Sun F., Báez-Díaz C., Sánchez-Margallo F.M. (2017). Canine prostate models in preclinical studies of minimally invasive interventions: Part II, benign prostatic hyperplasia models. Transl. Androl. Urol..

[3] Gobello C., Corrada Y. (2002). Noninfectious Prostatic Diseases in Dogs. Compend. Contin. Educ. Pract. Vet..

[4] Krawiec D.R., Heflin D. (1992). Study of prostatic disease in dogs: 177 cases (1981–1986). J. Am. Vet. Med. Assoc..

[5] Cunto M., Ballotta G., Zambelli D. (2022). Benign prostatic hyperplasia in the dog. Anim. Reprod. Sci..

[6] Romagnoli S., Krekeler N., de Cramer K., Kutzler M., McCarthy R., Schaefer-Somi S. (2024). WSAVA guidelines for the control of reproduction in dogs and cats. J. Small Anim. Pract..

[7] Zekraoui O., Bhojani N., Zorn K.C., Elterman D., Chughtai B. (2025). Management and Treatment of Benign Prostatic Hyperplasia Symptoms: Current Insights. Res. Rep. Urol..

[8] Roehrborn C.G., Bartsch G., Kirby R., Andriole G., Boyle P., de la Rosette J., Perrin P., Ramsey E., Nordling J., De Campos Freire G. (2001). Guidelines for the diagnosis and treatment of benign prostatic hyperplasia: A comparative, international overview. Urology.

[9] Barry M.J., Fowler F.J., O’Leary M.P., Bruskewitz R.C., Holtgrewe H.L., Mebust W.K., Cockett A.T. (1992). The American Urological Association symptom index for benign prostatic hyperplasia. The Measurement Committee of the American Urological Association. J. Urol..

[10] Zambelli D., Cunto M., Gentilini F. (2012). Validation of a model to develop a symptom index for benign prostatic hyperplasia in dogs. Reprod. Domest. Anim. Zuchthyg..

[11] Nizanski W., Ochota M., Fontaine C., Pasikowska J. (2020). Comparison of Clinical Effectiveness of Deslorelin Acetate and Osaterone Acetate in Dogs with Benign Prostatic Hyperplasia. Animals.

[12] Posastiuc F.P., Constantin N.T., Domain G., Spanoghe L., Van Soom A., Diaconescu A.I., Codreanu M.D. (2025). Is Canine Prostate-Specific Esterase a Reliable Marker for Benign Prostatic Hyperplasia Progression in Dogs?. Animals.

[13] Sannamwong N., Saengklub N., Sriphuttathachot P., Ponglowhapan S., England G., Kutzler M., Comizzoli P., Nizanski W., Rijsselaere T., Concannon P. (2012). Formula derived prostate volume determination of normal healthy intact dogs in comparison to dogs with clinical BPH. Proceedings of the 7th International Symposium on Canine and Feline Reproduction, Whistler, BC, Canada, 26–29 July 2012.

[14] Blum M.D., Bahnson R.R., Lee C., Deschler T.W., Grayhack J.T. (1985). Estimation of canine prostatic size by in vivo ultrasound and volumetric measurement. J. Urol..

[15] Ruel Y., Barthez P.Y., Mailles A., Begon D. (1998). Ultrasonographic evaluation of the prostate in healthy intact dogs. Vet. Radiol. Ultrasound Off. J. Am. Coll. Vet. Radiol. Int. Vet. Radiol. Assoc..

[16] Johnston S.D., Kamolpatana K., Root-Kustritz M.V., Johnston G.R. (2000). Prostatic disorders in the dog. Anim. Reprod. Sci..

[17] Smith J. (2008). Canine prostatic disease: Areview of anatomy, pathology, diagnosis, and treatment. Theriogenology.

[18] Berry S.J., Strandberg J.D., Saunders W.J., Coffey D.S. (1986). Development of canine benign prostatic hyperplasia with age. Prostate.

[19] Lévy X., Nizanski W., von Heimendahl A., Mimouni P. (2014). Diagnosis of common prostatic conditions in dogs: An update. Reprod. Domest. Anim..

[20] Mukaratirwa S., Chitura T. (2007). Canine subclinical prostatic disease: Histological prevalence and validity of digital rectal examination as a screening test. J. S. Afr. Vet. Assoc..

[21] Pinheiro D., Machado J., Viegas C., Baptista C., Bastos E., Magalhaes J., Pires M.A., Cardoso L., Martins-Bessa A. (2017). Evaluation of biomarker canine-prostate specific arginine esterase (CPSE) for the diagnosis of benign prostatic hyperplasia. BMC Vet. Res..

[22] Holst B.S., Holmroos E., Friling L., Hanas S., Langborg L.M., Franko M.A., Hansson K. (2017). The association between the serum concentration of canine prostate specific esterase (CPSE) and the size of the canine prostate. Theriogenology.

[23] Melandri M., Alonge S. (2021). Highlights on the Canine Prostatic Specific Esterase (CPSE): A diagnostic and screening tool in veterinary andrology. Vet. Med. Sci..

[24] Holst B.S., Carlin S., Fouriez-Lablée V., Hanås S., Ödling S., Langborg L.M., Ubhayasekera S., Bergquist J., Rydén J., Holmroos E. (2021). Concentrations of canine prostate specific esterase, CPSE, at baseline are associated with the relative size of the prostate at three-year follow-up. BMC Vet. Res..

[25] Albouy M., Sanquer A., Maynard L., Eun H.M. (2008). Efficacies of osaterone and delmadinone in the treatment of benign prostatic hyperplasia in dogs. Vet. Rec..

[26] Polisca A., Orlandi R., Troisi A., Brecchia G., Zerani M., Boiti C., Zelli R. (2013). Clinical efficacy of the GnRH agonist (deslorelin) in dogs affected by benign prostatic hyperplasia and evaluation of prostatic blood flow by Doppler ultrasound. Reprod. Domest. Anim. Zuchthyg..

[27] Sirinarumitr K., Johnston S.D., Kustritz M.V., Johnston G.R., Sarkar D.K., Memon M.A. (2001). Effects of finasteride on size of the prostate gland and semen quality in dogs with benign prostatic hypertrophy. J. Am. Vet. Med. Assoc..

