# Supplementary material for: Grading Canine Benign Prostatic Hyperplasia: A Survey of Veterinary Practice and the Development of a Web-Based Symptom Index
Source: Animals (Basel). 2026 Sep 9;16(18):2833. doi: 10.3390/ani16182833 (PMC13603634; doi:10.3390/ani16182833)
Supplement: Supplementary file 1 [file animals-16-02833-s001.zip › animals-4527747-supplementary.pdf]

# Supplementary Materials

## Clinical case used to evaluate respondents’ ability to assess BPH severity

Four clinical scenarios were presented to the webinar participants to evaluate respondents’ ability to assess BPH severity. For each case, participants were provided with the dog’s signalment (breed, age, and body weight), relevant clinical signs, serum CPSE concentration, ultrasonographic prostate dimensions, expected and measured prostate volumes, and representative ultrasonographic images. Based on the information provided, respondents were asked to classify each case into one of four categories: normal prostate, asymptomatic BPH, moderate BPH, or severe BPH.

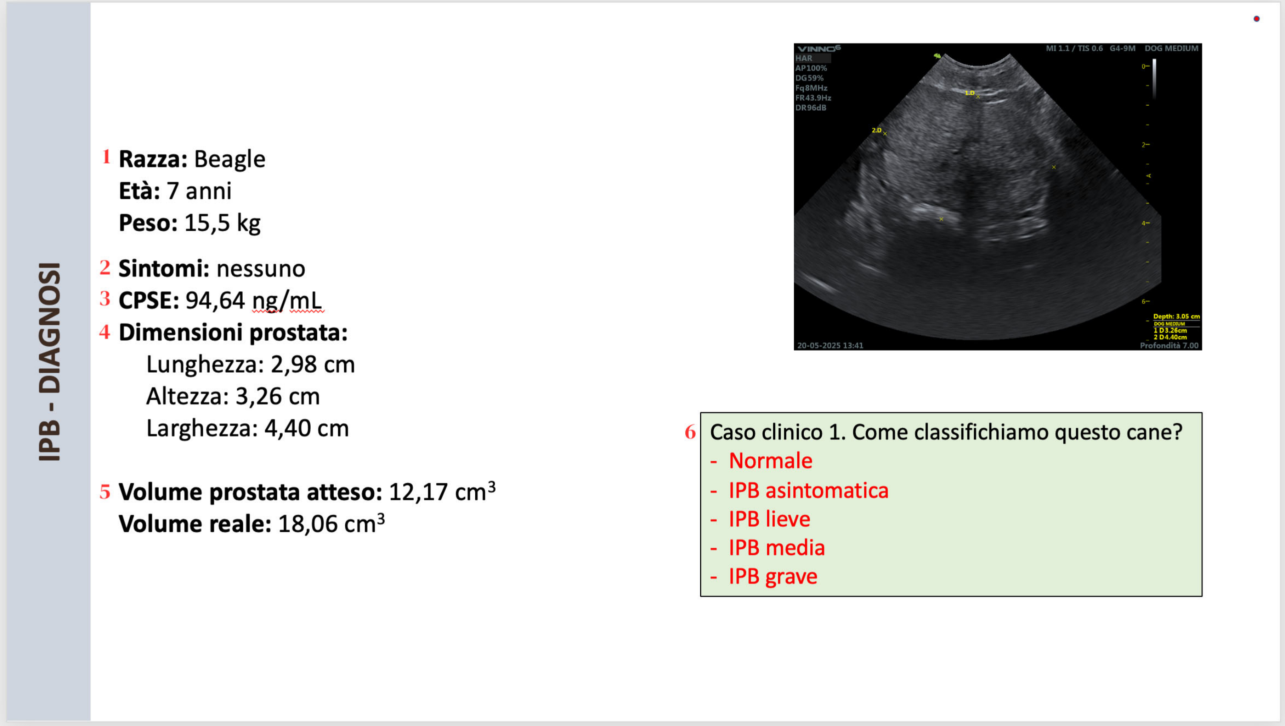


Figure S1: Original slide related to Clinical Case 1 used to evaluate respondents’ ability to assess. Since the webinar was in Italian, the slides are in Italian; for clarity, the main elements shown in the figure are translated in the accompanying legend: 1 - the dog’s signalment: Breed = Razza, Age = Età, Weight = Peso; 2 - Symptoms; 3 - serum CPSE concentration; 4 - ultrasonographic prostate dimensions; 5 - expected and measured prostate volumes, respectively Expected prostate volume and Actual volume; 6 - box. With the question and the four possible answers: “How do we classify this dog? normal prostate, asymptomatic BPH, moderate BPH, or severe BPH”.

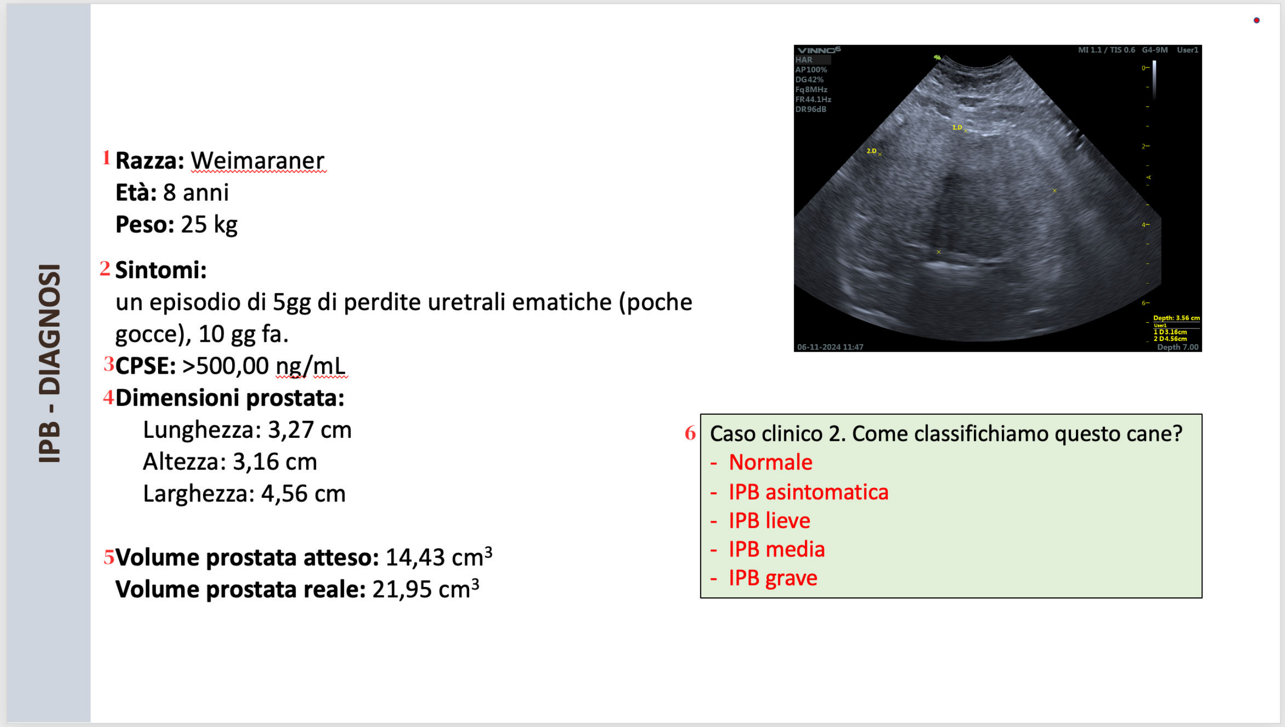


Figure S2: Original slide related to Clinical Case 2 used to evaluate respondents 'ability to assess. Since the webinar was in Italian, the slides are in Italian; for clarity, the main elements shown in the figure are translated in the accompanying legend: 1 - the dog's signalment: Breed = Razza, Age = Età, Weight = Peso; 2 - Symptoms; 3 - serum CPSE concentration; 4 - ultrasonographic prostate dimensions; 5 - expected and measured prostate volumes, respectively Expected prostate volume and Actual volume; 6 - box. With the question and the four possible answers: "How do we classify this dog? normal prostate, asymptomatic BPH, moderate BPH, or severe BPH".

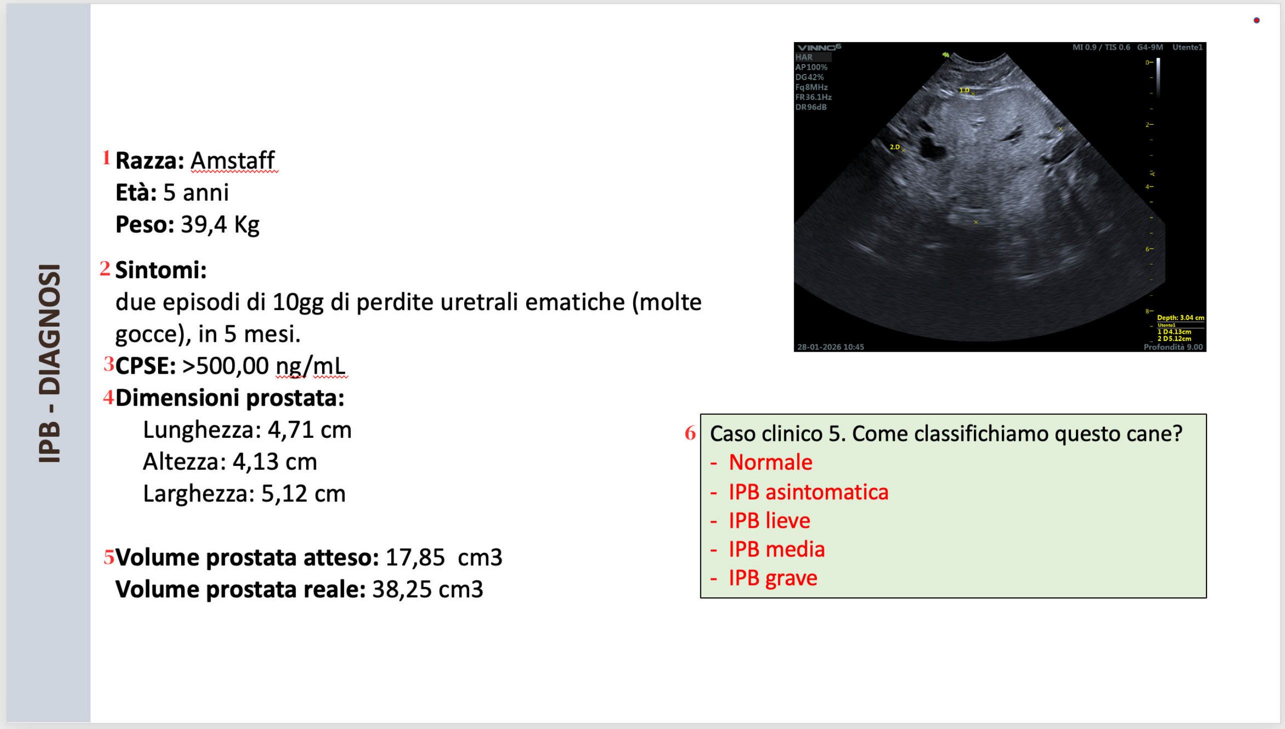


Figure S3: Original slide related to Clinical Case 3 used to evaluate respondents 'ability to assess. Since the webinar was in Italian, the slides are in Italian; for clarity, the main elements shown in the figure are translated in the accompanying legend: 1 - the dog's signalment: Breed = Razza, Age = Età, Weight = Peso; 2 - Symptoms; 3 - serum CPSE concentration; 4 - ultrasonographic prostate dimensions; 5 - expected and measured prostate volumes, respectively Expected prostate volume and Actual volume; 6 - box. With the question and the four possible answers: "How do we classify this dog? normal prostate, asymptomatic BPH, moderate BPH, or severe BPH".

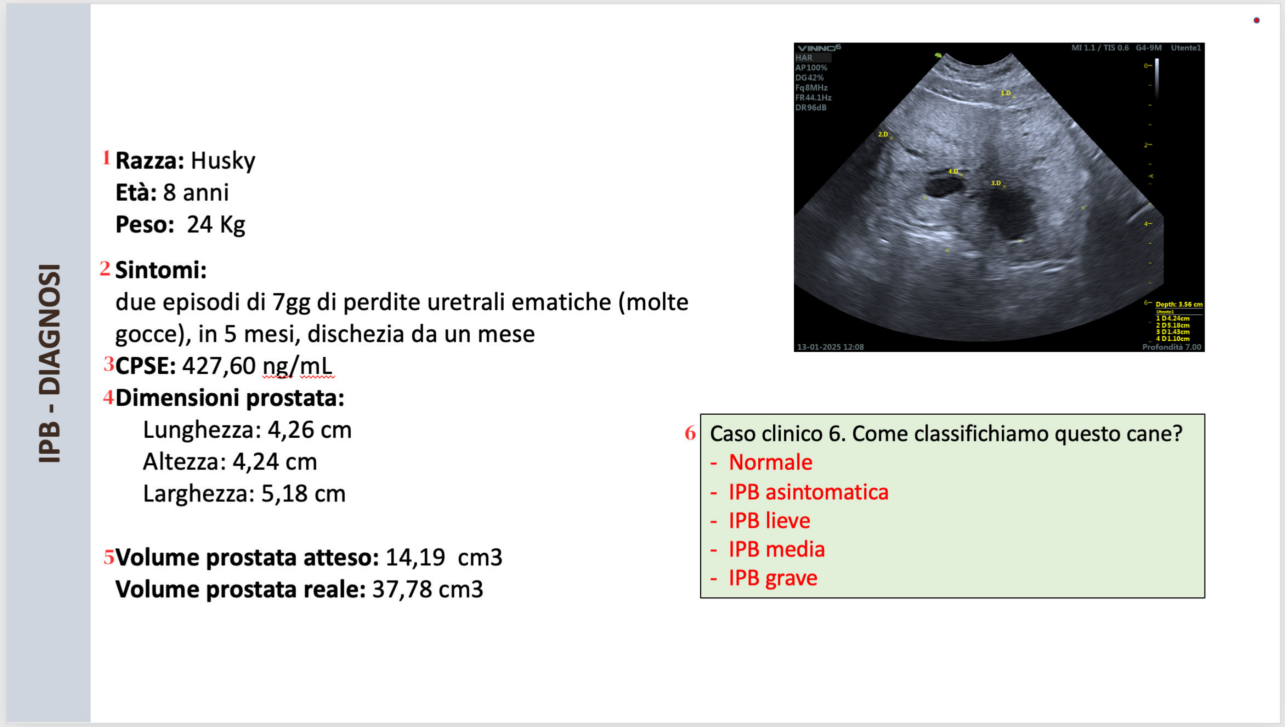


Figure S4: Original slide related to Clinical Case 4 used to evaluate respondents 'ability to assess. Since the webinar was in Italian, the slides are in Italian; for clarity, the main elements shown in the figure are translated in the accompanying legend: 1 - the dog's signalment: Breed = Razza, Age = Età, Weight = Peso; 2 - Symptoms; 3 - serum CPSE concentration; 4 - ultrasonographic prostate dimensions; 5 - expected and measured prostate volumes, respectively Expected prostate volume and Actual volume; 6 - box. With the question and the four possible answers: "How do we classify this dog? normal prostate, asymptomatic BPH, moderate BPH, or severe BPH".

# Clinical case used to evaluate respondents’ therapeutic decision-making

Four clinical scenarios were presented to the webinar participants to evaluate respondents’ therapeutic decision-making. For each case, participants were provided with the dog’s signalment (breed, age, and body weight), relevant clinical signs, serum CPSE concentration, ultrasonographic prostate dimensions, expected and measured prostate volumes, and representative ultrasonographic images. Based on the information provided, respondents were asked to select the therapeutic approach they considered most appropriate: Surgical therapy (orchiectomy), pharmacological therapy, androgen deprivation pharmacological therapy, or no therapy.

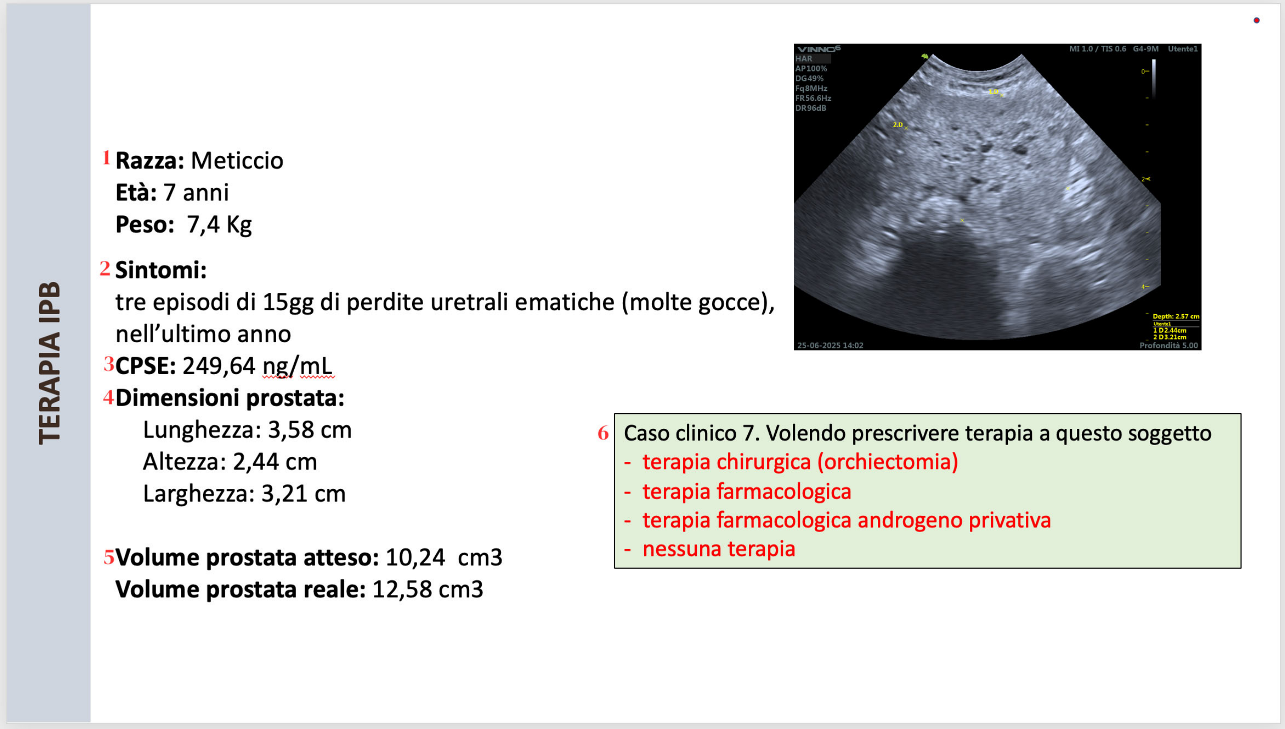


Figure S5: Original slide related to Clinical Case 1 used to evaluate respondents ’therapeutic decision-making. Since the webinar was in Italian, the slides are in Italian; for clarity, the main elements shown in the figure are translated in the accompanying legend: 1 - the dog’s signalment: Breed = Razza, Age = Età, Weight = Peso; 2 - Symptoms; 3 - serum CPSE concentration; 4 - ultrasonographic prostate dimensions; 5 - expected and measured prostate volumes, respectively Expected prostate volume and Actual volume; 6 - box with the question and the four possible answers: If you were to prescribe therapy for this individual, what would you prescribe? Surgical therapy (orchiectomy), pharmacological therapy, androgen deprivation pharmacological therapy, or no therapy.

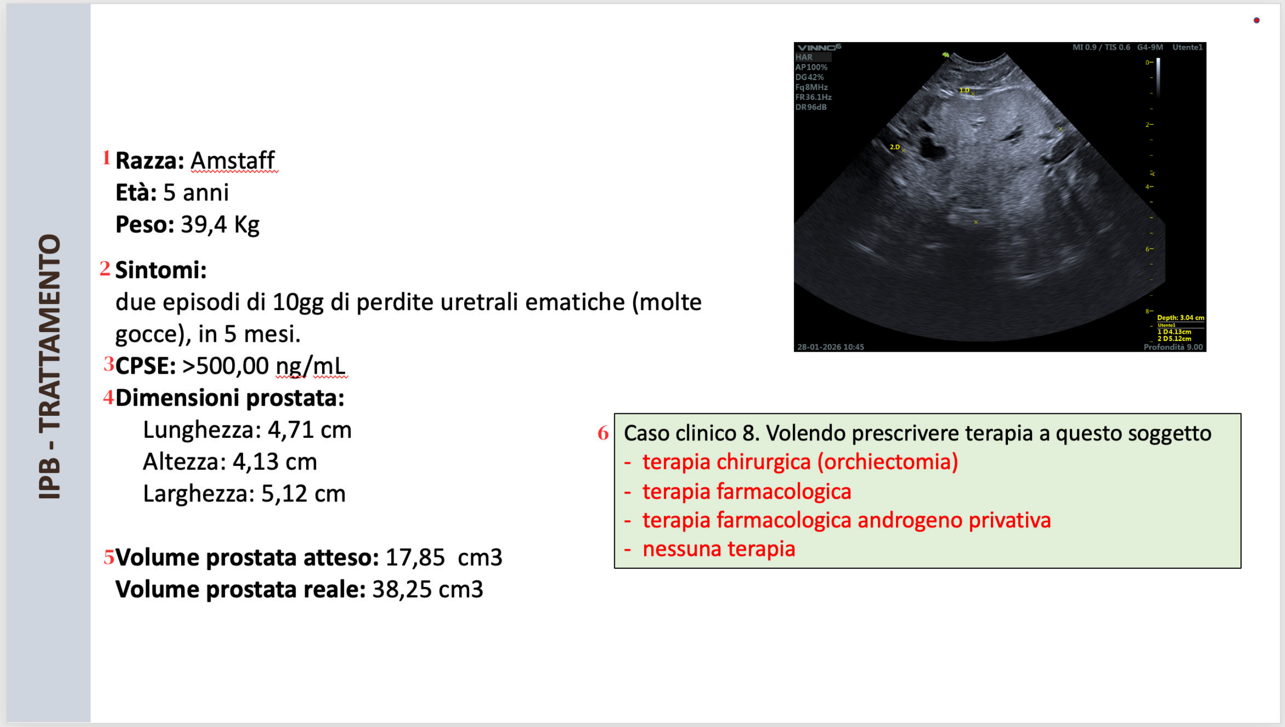


Figure S6: Original slide related to Clinical Case 2 used to evaluate respondents 'ability to assess. Since the webinar was in Italian, the slides are in Italian; for clarity, the main elements shown in the figure are translated in the accompanying legend: 1 - the dog's signalment: Breed = Razza, Age = Età, Weight = Peso; 2 - Symptoms; 3 - serum CPSE concentration; 4 - ultrasonographic prostate dimensions; 5 - expected and measured prostate volumes, respectively Expected prostate volume and Actual volume; 6 - box with the question and the four possible answers: If you were to prescribe therapy for this individual, what would you prescribe? Surgical therapy (orchiectomy), pharmacological therapy, androgen deprivation pharmacological therapy, or no therapy.

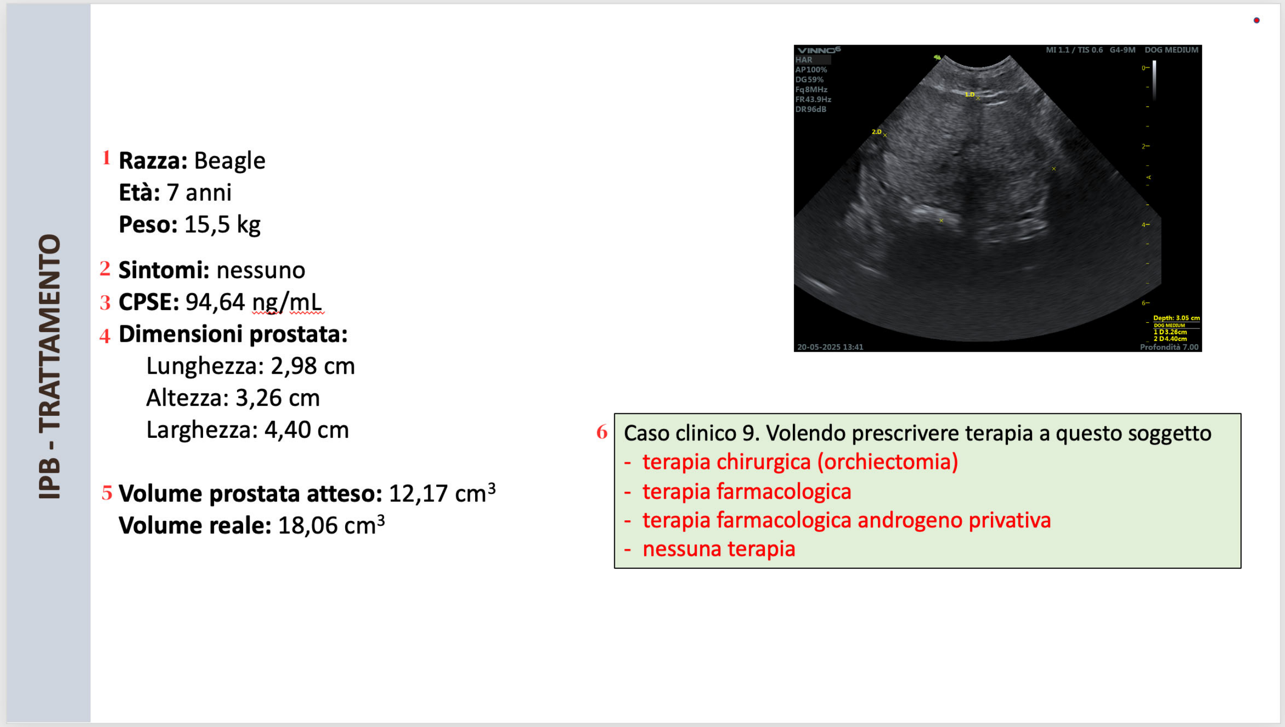


Figure S7: Original slide related to Clinical Case 3 used to evaluate respondents 'ability to assess. Since the webinar was in Italian, the slides are in Italian; for clarity, the main elements shown in the figure are translated in the accompanying legend: 1 - the dog's signalment: Breed = Razza, Age = Età, Weight = Peso; 2 - Symptoms; 3 - serum CPSE concentration; 4 - ultrasonographic prostate dimensions; 5 - expected and measured prostate volumes, respectively Expected prostate volume and Actual volume; 6 - box with the question and the four possible answers: If you were to prescribe therapy for this individual, what would you prescribe? Surgical therapy (orchiectomy), pharmacological therapy, androgen deprivation pharmacological therapy, or no therapy.

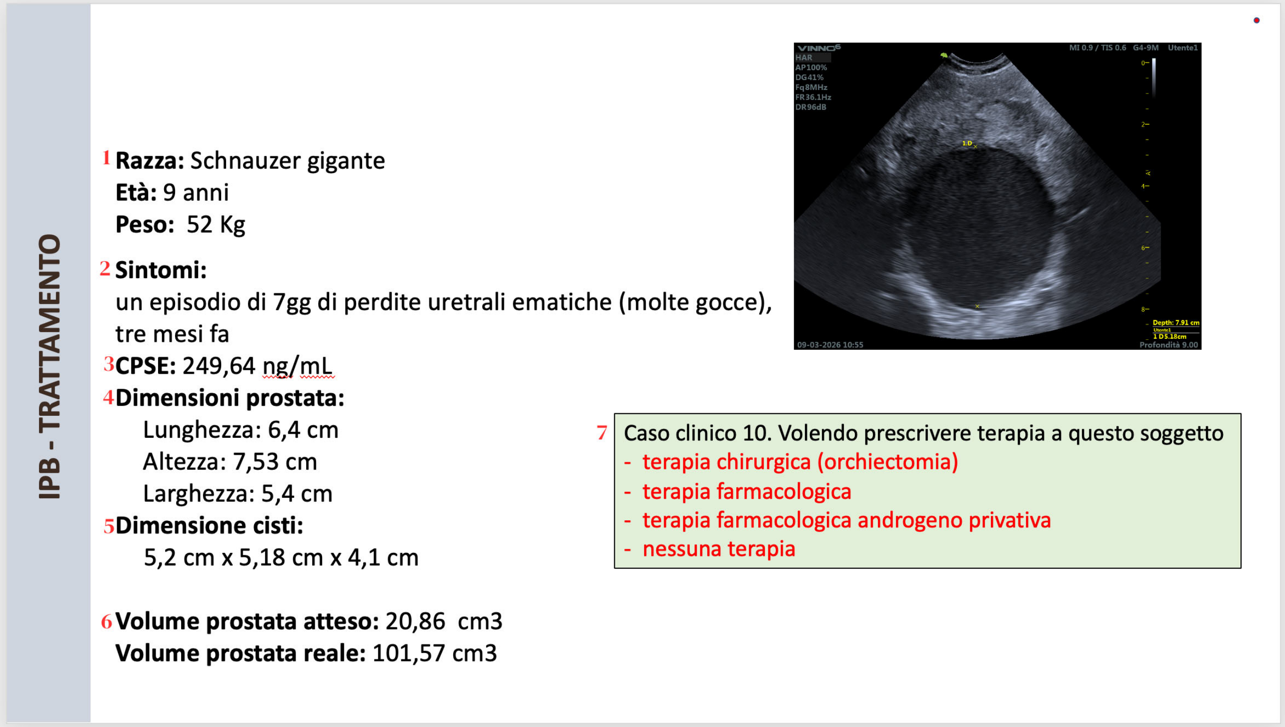


Figure S8: Original slide related to Clinical Case 4 used to evaluate respondents 'ability to assess. Since the webinar was in Italian, the slides are in Italian; for clarity, the main elements shown in the figure are translated in the accompanying legend: 1 - the dog's signalment: Breed = Razza, Age = Età, Weight = Peso; 2 - Symptoms; 3 - serum CPSE concentration; 4 - ultrasonographic prostate dimensions; 5 - Prostatic cyst dimension; 6 - expected and measured prostate volumes, respectively Expected prostate volume and Actual volume; 7 - box with the question and the four possible answers: If you were to prescribe therapy for this individual, what would you prescribe? Surgical therapy (orchiectomy), pharmacological therapy, androgen deprivation pharmacological therapy, or no therapy.
